# Epigenetic inheritance of gene-silencing is maintained by a self-tuning mechanism based on resource competition

**DOI:** 10.1016/j.cels.2022.12.003

**Published:** 2023-01-18

**Authors:** Omer Karin, Eric A. Miska, Benjamin D. Simons

**Affiliations:** 1 Department of Applied Mathematics and Theoretical Physics, Centre for Mathematical Sciences, University of Cambridge, Cambridge, CB3 0WA, UK; 2 Wellcome Trust/Cancer Research UK Gurdon Institute, University of Cambridge, Cambridge, CB2 1QN, UK; 3 Department of Mathematics, Imperial College London, London, SW7 2AZ, UK; 4 Wellcome Trust-Medical Research Council Cambridge Stem Cell Institute, Jeffrey Cheah Biomedical Centre, University of Cambridge, Cambridge, CB2 0AW, UK

**Keywords:** Epigenetic memory, transgenerational inheritance, biological memory, systems biology, mathematical modelling, self-tuning to criticality

## Abstract

Biological systems can maintain memories over long timescales, with examples including memories in the brain and immune system. It is unknown how functional properties of memory systems, such as memory persistence, can be established by biological circuits. To address this question, we focus on transgenerational epigenetic inheritance in C. elegans. In response to a trigger, worms silence a target gene for multiple generations, resisting strong dilution due to growth and reproduction. Silencing may also be maintained indefinitely upon selection according to silencing levels. We show that these properties imply fine-tuning of biochemical rates in which the silencing system is positioned near the transition to bistability. We demonstrate that this behavior is consistent with a generic mechanism based on competition for synthesis resources, which leads to self-organization around a critical state with broad silencing timescales. The theory makes distinct predictions and offers insights into the design principles of long-term memory systems.

A record of this paper’s Transparent Peer Review process is included in the Supplemental Information.

## Introduction

The ability to record and maintain memories is one of the distinctive and most remarkable features of living systems. Examples include the ability of the brain to record past experiences, the immune system to remember pathogen encounters, and many organisms to transfer epigenetic information across generations^1–6^.

**Figure 1:**
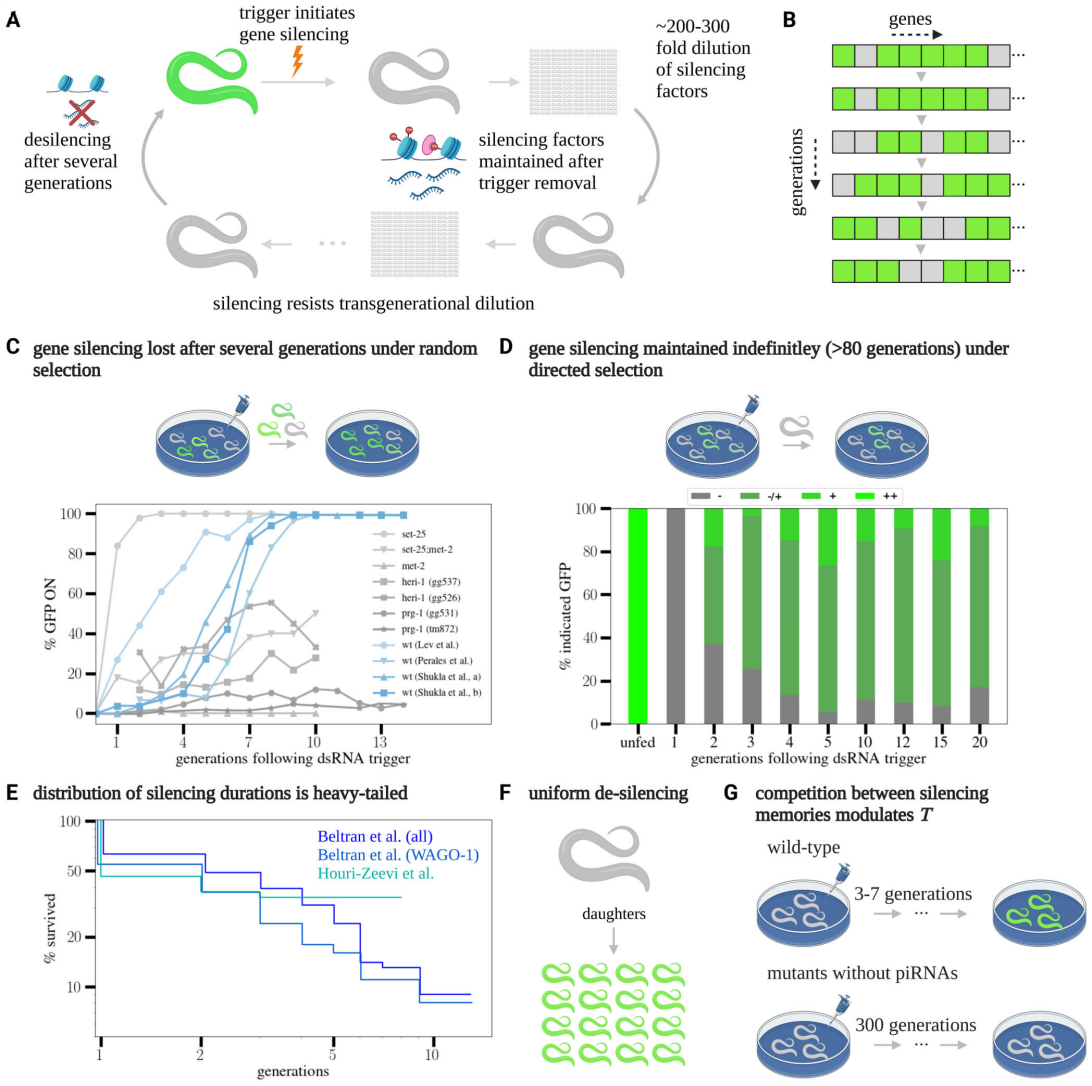
Experimental observations of transgenerational gene silencing in C. elegans constrain possible response mechanisms. (A) Transgenerational co-transcriptional silencing of *gfp* expression in *C. elegans* is initiated following the presentation of a dsRNA trigger and can be tracked at the level of individual animals. Silencing is associated with production, transmission, and maintenance of siRNAs and silencing chromatin marks (H3K9me3) that persists over multiple generations, resisting many-fold dilution due to reproduction of worms. Silencing terminates following the loss of the relevant siRNA molecules and silencing chromatin modifications. (B) Schematic illustrating that stochastic silencing (depicted as a transition from color gray) also occurs in many endogenous genes and may persist over the timescale of multiple generations. (C) Percentage of worms that show *gfp* expression as a function of generation number following induced silencing. In the wild-type (wt), silencing persists for around *T* ≈ 3 − 7 generations, when averaged over the population of worms. (Blue lines denote wt strains, data from^18,19,25^; note that Shukla et al., a,b refer to measurements on wt strains from Figure S1A,B). However, some mutant strains show altered average silencing times that can be shorter, such as the *set-25* knockdown where *T* ≈ 1 generation, or longer, such as the *met-2* knockdown where *T* ≳ 30 generations. (Gray lines denote mutant strains, data from ^18,19,25^). (D) Although the average persistence time of *gfp* silencing across generations is limited, if worms with the highest degree of silencing are continuously selected from the ensemble at each generation, silencing levels of their progenies converge towards a broad and stationary distribution that can be maintained indefinitely (panel adapted from Figure 1 of ^20^; ++ corresponds to strong *gfp* signal, while +, +/-, and – correspond to increasingly weaker *gfp* signal). (E) Distribution of silencing durations for a given lineage is “heavy-tailed”, showing a slow (power-law like) decay at long silencing times (data on *gfp* silencing in individual lineages from ^16^ (turquoise), data on siRNA epimutation duration from ^10^ (shades of blue). (F) Houri-Zeevi et al. showed that de-silencing of *gfp* is irreversible and occurs uniformly amongst sister worms^16^ – that is, when a worm becomes irreversibly de-silenced, all (or nearly all) sisters also become de-silenced. (G) Amplification and maintenance of silencing machinery (e.g., siRNAs) is limited by shared synthesis components. Resource competition is tightly associated with silencing duration, and mutants where competition between silencing memories is modulated (e.g., animals that lack piRNAs) have much longer silencing durations.

From the perspective of systems biology, it is important to understand how the functional properties of long-term memory systems can be implemented by biological interactions, such as networks of biochemical reactions. This challenge is exemplified by transgenerational transcriptional silencing in *C. elegans* worms. When exposed to a double-stranded RNA (dsRNA) trigger, worms will silence its homologous gene. Remarkably, when expressed in the germline, this silencing can persist over multiple generations even in the absence of the original trigger^7^. Transgenerational silencing also occurs in response to various endogenous triggers^2,8–10^. At the molecular level, there are two processes that contribute to silencing – the production and amplification of 22-nucleotide-long short-interfering RNAs (22G siRNAs) and the deposition of silencing histone modifications including H3K9me3^4,11^ (Figure 1A, B). Transcriptional silencing allows *C. elegans* to protect itself from foreign agents such as viruses^12^ and transposable elements ^13^, and to transmit environmental information across generations^9,14^. The relatively short generation time of *C. elegans*, together with the ability to control memory induction, makes it a particularly attractive model to study memory at both the molecular^15^ and phenomenological^10,16^ level.

The goal of this study is to make use of the known properties of this system to address two basic and generic questions: (1) How can a biological memory system establish and tune a prolonged time of memory duration? (2) Are there features of the memory system that are beneficial for retaining relevant memories? Both questions are functionally relevant for the epigenetic silencing system. Silencing in wild-type worms is retained for *T* ≈ 3 − 7 generations following the removal of the trigger, which is much longer than the typical timescale of dilution of the silencing factors by growth and reproduction^17^, yet much shorter than the silencing durations observed in certain mutants^18,19^. Tuning the timescale *T* allows the system to balance the potential benefits of long-term silencing with the aberrant accumulation of possibly irrelevant silencing memories. Additionally, there is variation in silencing levels between individual silenced worms, and experimental selection on this variation allows silencing to be retained indefinitely in the population^20^, implying that the variation is heritable. It is unclear how these features (tuning of memory duration over prolonged timescales, and heritable variation that allows for selection) can be implemented within the framework of a biochemical network.

Here, in this theoretical study, we show that the functional features of the epigenetic silencing system in *C. elegans* are consistent with a competition mechanism that maintains the dynamics of silencing memories near a transition to bistability. To develop this framework, we first summarize the key existing experimental results on transgenerational epigenetic inheritance of silencing in *C. elegans*, focusing on the silencing of the *gfp* transgene. These findings are then used to motivate and constrain a minimal model, where 22G-siRNA amplification is governed by a bistable switch, and there is negative feedback due to the deposition of silencing chromatin marks. By itself, this circuit can be either bistable (transcription is ON/OFF) or provide a monostable decaying response (once activated, silencing factors decay to a stable unsilenced state). We then show that a wide range of experimental findings are consistent with a biological circuit that is excitable and fine-tuned to the vicinity of a transition to bistability, raising the question of how such fine-tuning can be achieved in a biological setting. To address this, we note that silencing memories are not maintained in isolation, but rather share various synthesis components such as RNA-dependent RNA polymerases (RdRP) and Argonaute (AGO) proteins. Competition for these resources engages the collective behavior of many silencing memories, where their accumulation reduces the available amplification capacity. We show that, when accounting for such competition, the dynamics of silencing memories becomes self-organized in a narrow range near the transition to bistability in a manner that provides a tunable delay time, whose magnitude may be much longer than the timescale of the turnover of the underlying molecular components. Within this framework, the delay time is robust to noise and fluctuations in circuit parameters. Self-organization near the transition to bistability results in large, static, variation of gene silencing levels between individual worms, allowing for selection according to silencing levels. Finally, we argue that the memory model provides a unifying explanation of multiple existing – often paradoxical – observations on the transcriptional silencing of exogenous and endogenous targets in *C. elegans*, and we propose a series of new experiments that could be used to test the model. Based on the simplicity of the model assumptions, we postulate that this mechanism of *self-tuned criticality by competition* may prevail in other memory systems. We therefore conclude by outlining the general hallmarks and wider predictions of the model.

## Results

### Key experimental observations from prior studies constrain models for transgenerational epigenetic inheritance

A well-established system for studying transgenerational inheritance involves the silencing of a constitutively expressed *gfp* reporter gene^2,8,16,17,20–22^. Silencing of a *gfp* reporter provides a quantitative proxy for the degree of silencing in an individual animal, and how silencing evolves in a population over generations. This approach has been used extensively to explore the influence on inheritance dynamics of selection^16,20,23^ and genetic background^18,19,24–27^. *gfp* silencing experiments show that, following its induction, silencing is typically reversed in wild-type animals within around *T* ≈ 3 − 7 generations (Figure 1C). Moreover, mutant strains can show altered inheritance patterns, including long-term silencing that can persist for tens of generations or more (Figure 1C).

Most transgenerational silencing experiments (including those reproduced in Figure 1C) involve the transfer of random progeny to new plates at each generation, with the population of worms at each generation showing various degrees of silencing. However, Vastenhouw et al. found that, by selecting worms that show the highest degree of silencing at each generation, a stable inheritance pattern of silencing is established among progenies that can last for at least 80 generations^20^ (Figure 1D). Notably, in every generation, the same continuous distribution of silencing states is replicated or re-established. These findings suggest that the silencing of a target gene that provides a selective benefit to an organism can be maintained stably in the population.

Further quantitative insight into transgenerational inheritance dynamics comes from a recent study by Houri-Zeevi et al., where inheritance was tracked in multiple lineages composed of more than 20,000 worms^16^. They showed that some individual lineages retain silencing for much longer than the average duration time *T* of the population, as indicated by the distribution of silencing times that show a “heavy-tail” marked by a power-law-like dependence (Figure 1E, see Figure S1 for a log-linear plot). A similar heavy-tailed distribution has been observed in the duration of silencing epimutations in endogenous genes, which occur following stochastic initiation triggers (Figure 1E). Their study also revealed that, in contrast to the variability in silencing levels observed at the population level by Vastenhouw et al. under selection, the process of de-silencing occurs uniformly in the descendants of each worm (Figure 1F).

Silencing duration times also appear to be modulated by resource competition (Figure 1G). The amplification of siRNAs requires the synthesis of resources such as AGO proteins and RdRP complexes. As these are shared between many small RNA molecules, they limit the overall synthesis rate, leading to competition for resources. Evidence for such competition is extensive in *C. elegans*^17,27–32^, and experiments where long-lasting inheritance of silencing was demonstrated were accompanied by large-scale global changes in siRNAs^18,19^. Specifically, a recent study demonstrated that, in the absence of piRNAs (a major source of endogenous small RNAs that compete with siRNAs over shared synthesis resources), silencing can persist for hundreds of generations (Figure 1F)^19^.

### A model for transgenerational epigenetic inheritance based on interactions between siRNAs and silencing chromatin marks

**Figure 2:**
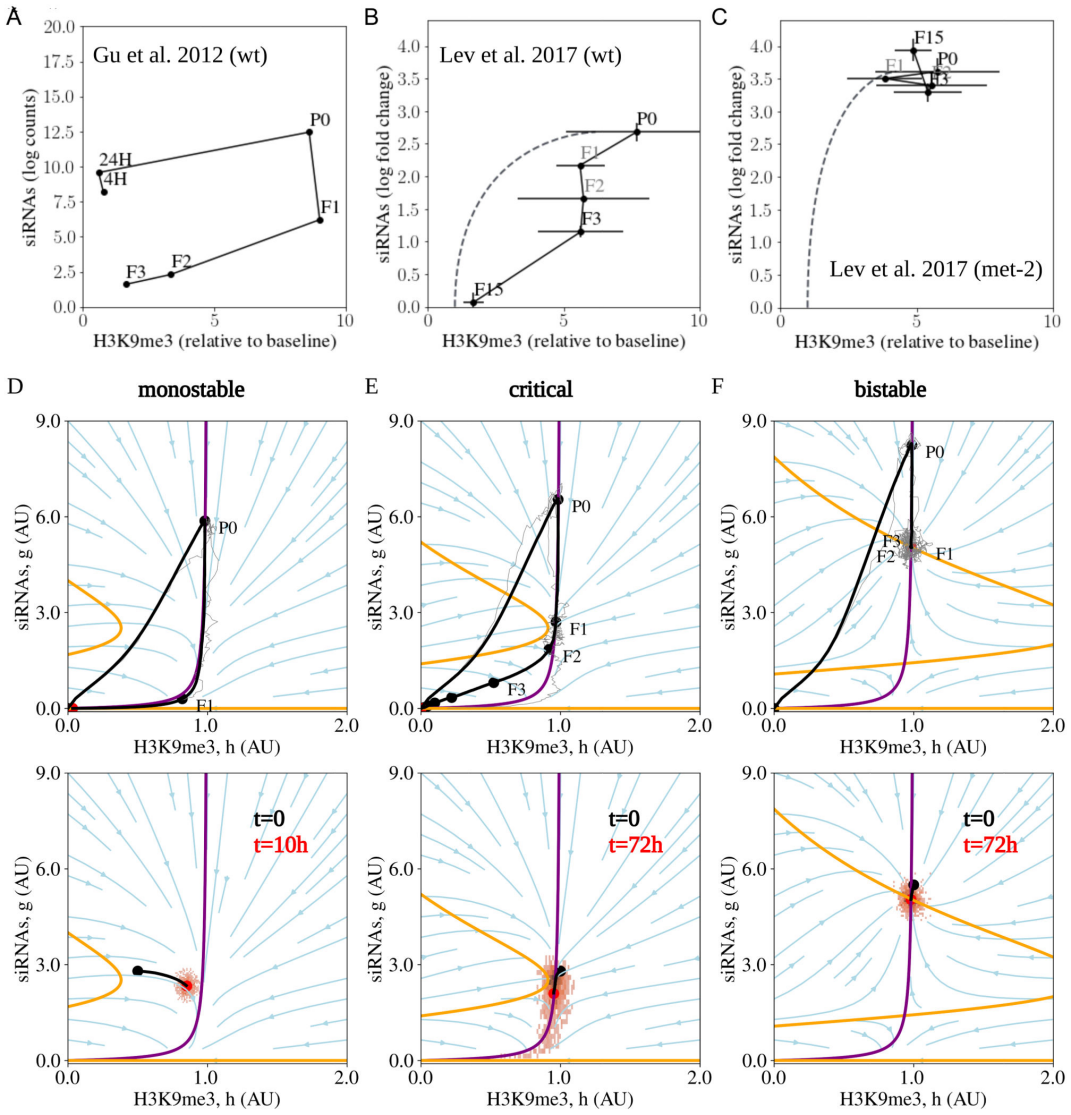
Toggle-Inhibitor (TI) model captures transgenerational dynamics of gene silencing factors. (A-C) Measured transgenerational dynamics of target-specific siRNAs and the accumulation of H3K9me3 marks on the target gene: in (A) wild-type (*wt*) worms following *lin-15B* dsRNA trigger (data from ^33^); and (B) in *wt* worms and (C) *met*-2 worms following *gfp* dsRNA trigger (data from ^18^). Note that siRNA data was not available for F1 and F2 in panels B and C, and so was imputed using measured P0 and F3 siRNA levels. Measurements of H3K9me3 were performed using ChIP assays, while siRNA counts were quantified using siRNA-seq (see ^18,33^ for details. Error bars denote SEM of three repeats for Lev et al). (D-F) Phase plane representations of the noise-free *TI* model in the three regimes: (D) monostable, (E) near saddle-node bifurcation or critical, and (F) bistable. Nullclines for $\dot{h}=0$ and $\dot{g}=0$ are shown as lines, parameterized by $h=\frac{\psi }{\gamma_{2}}\frac{g}{k_{3}+g}$ (purple) and $h=k_{2}\left(\frac{V}{\gamma_{1}}\frac{g^{n-1}}{g^{n}+k_{1}^{n}}-1\right)$, *g* = 0 (orange), respectively. The model has a stable unsilenced state at the origin while, for appropriate parameters, a silenced stable state (at high *g*, *h*) can emerge. The upper panels depict the locus of trajectories of *g* and *h* obtained from stochastic simulations of the *TI* model with noise averaged over the population (black lines, average over *n* = 1000 instances), as well as an example of an individual worm lineage (thin gray line). The lower panels depict the distribution of end states after one generation or less, starting from a single ancestral state positioned at the given point in phase space (black dot), representing the evolution over time of the silencing state (red dot represents the center-of-mass of the distribution of end states). Note that, in the monostable regime, the coordinates of all progenies are remote from the parent, moving towards the de-silencing state. Simulation parameters are provided in Table S1.

Taken together, these experimental findings (summarized in Figure 1, and illustrated schematically in Figure S2) represent the major hallmarks of gene silencing dynamics in *C. elegans* and constitute a set of exacting and diverse constraints on the underlying mechanisms that regulate this silencing memory system. Here, we focused on using these observations to identify how the functional properties of the epigenetic memory system may arise from interactions of the underlying biochemical components. Specifically, we placed emphasis on the two core molecular components of transgenerational inheritance: the concentration of the target-specific secondary siRNA pool (22G siRNAs) and the concentration of H3K9me3 on the target gene, both of which can be measured using small RNA sequencing (sRNAseq) and chromatin immunoprecipitation followed by sequencing (ChIPseq), respectively (Figure 2). We considered experiments where both quantities were measured in populations of worms following a dsRNA trigger (see Figure 2A, B for details)^18,33^. Following exposure of wild-type animals to the dsRNA trigger, the P0 generation, the concentration of the target-specific siRNA pool (denoted *g*) and concentration of H3K9me3 around the target gene (denoted *h*), in populations of whole animals, follow a specific trajectory in phase-space (Figure 2A, B): An initial jump in *g*, followed by an increase in *h*, establishes a high level of gene silencing in P0. In subsequent generations, *g* decays slowly, while complete de-silencing in later generations is accompanied by reversal of both *g* and *h* back to their baseline (pre-trigger) values, closing the trajectory. In the *met-2* knockout animals, which show stable silencing of *gfp* over many generations, high levels of *g* and *h* are maintained at an effective transgenerational “stable fixed point” (Figure 2C).

Altogether, the observed dynamics are consistent with a model of siRNAs and H3K9me3 with the following minimal set of processes (Figure 2D-F): (i) siRNAs amplify cooperatively^16,34^, resulting in bistability at low levels of H3K9me3; (ii) siRNAs promote the placement of H3K9me3 silencing marks^33,35^; (iii) H3K9me3 modifications induce inhibition of the siRNA pool, e.g., due to transcriptional inhibition, which depletes the template mRNA, or due to the recruitment of allosteric inhibitors^25^; and (iv) silencing factors decay on the transgenerational timescale by growth and reproduction. While it is possible to envisage additional mechanisms and processes acting on siRNAs and silencing marks, as well as other factors contributing to silencing, we propose that (i)-(iv) constitute the core set of processes that both capture the dynamics of siRNAs and H3K9me3 and, as we will show below, the observed transgenerational silencing features presented in Figure 1.

Mathematically, based on processes (i)-(iv), we propose that the dynamics of the siRNA pool and H3K9me3 concentration can be captured by the two coupled noisy rate equations:

(1) $$dg=\left(I\left(t\right)+V\frac{g^{n}}{k_{1}{}^{n}+g^{n}}\frac{k_{2}}{k_{2}+h}-\gamma_{1}g\right)dt+S_{1}\left(g,h\right)\;dW_{1}$$

(2) $$dh=\left(\psi \frac{g}{k_{3}+g}-\gamma_{2}h\right)dt+S_{2}\left(g,h\right)\;dW_{2}$$

Here, *I*(*t*) denotes a time-dependent pulse-like induction term representing the initial trigger arising from the ingestion of bacteria expressing dsRNA in P0. The second production term, describing the cooperative amplification of 22G siRNA, scales in proportion to $V\frac{g^{n}}{k_{1}{}^{n}+g^{n}}$, having a maximum amplification capacity *V* (that may be limited by available synthesis machinery), cooperativity *n*, and a half-maximal amplification activity at *g* = *k*_1_. The amplification term is modulated by an inhibitory factor, $\frac{k_{2}}{k_{2}+h}$, associated with silencing chromatin marks, with half-maximal strength at *h* = *k*_2_. 22G siRNAs stimulate the production of silencing chromatin marks with a maximum synthesis capacity *ψ* and half-way activity *g* = *k*_3_. We note that, on the transgenerational timescale, the production terms effectively combine both the production of silencing factors and their loading onto oocytes. Finally, *γ*_1_, *γ*_2_ denote the respective decay rates of *g*, *h* due to growth and reproduction. To account for stochastic fluctuations between individuals due to variations in growth rate, as well as fluctuations in the expression of relevant factors such as heat shock proteins ^16^, we have included noise terms *S*_1_(*g*, *h*) *dW*_1_, *S*_2_(*g*, *h*) *dW*_2_, where *W*_1_, *W*_2_ are Wiener processes ^36^. (Note that, for the stochastic simulations of the model, we consider multiplicative noise, setting *S*_1_(*g*, *h*) = *σ*_1_*g*., *S*_2_(*g*, *h*) = *σ*_2_*h*, with *σ*_1_, *σ*_2_ characterizing the strength of the noise.) Together, these coupled rate equations comprise the *Toggle-Inhibitor (TI)* model.

### The TI model has three regimes with distinct dynamics, with experimental observations consistent with fine-tuning around the critical regime

The *TI* model (Eqs. (1,2)) constitutes an excitable circuit that combines positive and negative feedback, a motif prevalent in many biological systems^37,38^. Due to cooperativity in the amplification of siRNAs, only a sufficiently large input trigger can elicit a durable silencing response, protecting the system from aberrant noise-induced silencing. This ensures that the *unsilenced* or *ON* state is stable against small fluctuations in siRNA expression. Once a response is initiated (for example, by ingestion of dsRNA), several dynamical trajectories in the phase-space of *g* and *h* are possible depending on parameter values (Figure 2D-F, see Movies S1, S2 for animations of trajectories for different regimes). Depending on the scale of the maximum amplification capacity *V*, it is possible to generate monostability (low *V*), bistability (high *V*), or an intermediate response when the system is positioned close to a saddle-node bifurcation (*V* ≈ *V*_crit_). This latter regime, which we denote as the “critical” regime, requires fine-tuning of *V* within a very narrow parameter range, a point to which we will return presently. These three regimes make distinct predictions about the nature of the transgenerational dynamics following a trigger.

In the following section, we will analyze each of the three regimes, and then compare the theoretical predictions with the experimental observations described in Figure 1. The arguments laid out in this section are detailed in the Model Comparison section of the Methods.

In the monostable regime (Figure 2D, corresponding to *V* < *V*_crit_), following dsRNA exposure, the model predicts an increase in *g* followed by an increase in *h*, after which both components decay according to the respective turnover rates *γ*_1_, *γ*_2_. Generalizations of the monostable model could include possible variation between individuals due to differences in turnover rates, or asymmetric partitioning of silencing factors across and between generations as a result, for example, of small number fluctuations. However, this latter scenario would seem to be unlikely given the approximate uniformity of de-silencing across siblings, as reported by ^16^. Transgenerationally, decay is likely to be governed by a characteristic dilution rate *γ*, which corresponds to the decay of silencing factors in the absence of autocatalysis. Since the silencing factors in each animal need to be partitioned between ~250 laid eggs, one can expect that, without autocatalysis, there would be a dilution by a factor of many orders of magnitude within a memory lifetime (estimated at around a factor of 250^4^ ≈ 4 ⋅ 10^9^ after 4 generations ^16^). This is consistent with the observation that the silencing of genes that are expressed only in the soma, and thus not amplified in the germ line, disappears after a single generation^24^. This implies that, in the monostable regime, we would expect $T\approx \frac{1}{r}\ll 1$ generation. Additionally, in the monostable regime, there is a monotonic progression of the dynamics in phase-space (Figure 2D), which as we will later discuss is consequential for the ability to perform effective selection.

We first question whether the inheritance system is positioned in the monostable regime. This is unlikely, for several reasons: First, as monostable decay would be governed by strong dilution of silencing factors over the transgenerational timescale, we would expect silencing duration of *T* = 1 generation, while silencing is observed to last *T* ≈ 3 − 7 generations. Moreover, some mutants, such as the *met-2* strain, and mutants lacking piRNAs, show stable silencing for many generations, translating to hundreds of generations for the latter^19^, requiring an even lower decay rate. Finally, in the monostable setting, selection according to silencing levels would be ineffective since variation is not static, but with each generation moves directionally in phase space (Figure 2D). Based on these considerations, monostability is an unlikely candidate to explain the range of experimental phenomenology (Figure 1).

We next analyzed the regime of bistability, considered a hallmark of biological memory systems ^39^ (Figure 2F, corresponding to *V* > *V*_crit_). Here, to describe the dynamics, one may think of a thermal analogy in which the silencing state is indexed by the position of a particle in a potential well, equilibrated through interaction with its surroundings. In a monostable system, the dsRNA trigger is akin to pushing the particle to a higher position in the potential well and allowing it to relax back to equilibrium. In a bistable system, the potential well has two degenerate minima corresponding, respectively, to the ON/OFF states. In this case, a trigger that is strong enough can transition the system between the stable ON state into the adjacent metastable OFF (silenced) state. This perturbation allows the system to maintain silencing for a timescale that can far exceed that of its individual components, providing a potential explanation for long-term silencing.

In the bistable regime, while the transition from OFF to ON occurs after the presentation of the dsRNA input stimulus, the reverse transition (ON to OFF) occurs spontaneously, long after the input trigger has been removed. To address how such spontaneous transitions occur, two explanations may be advanced: (1) there is variation in initial conditions or stable variation in parameters following the removal of the trigger (see, e.g., Houri-Zeevi et al.^16^); or (2) temporal fluctuations can spontaneously transition the system from ON to OFF, akin to effect of thermal fluctuations on chemical reactions. This can include fluctuations in *g,h*, as well as fluctuations in circuit parameters.

In the case of bistability with variation in initial conditions (e.g., a uniform initial distribution of *g* after triggering), the silencing state of some worms will move to the stable ON state while that of others will decay back to the stable OFF state. The former timescale could be very long, while the latter timescale is dominated by dilution and thus should decay rapidly within a single generation (see Methods). Thus, overall, variation in initial conditions in the bistable regime would result in a bimodal distribution of silencing durations. In contrast, the observed distribution of silencing durations is continuous (that is, many worm lineages retain silencing for only a few generations, Figure 1E). Additionally, such a model would imply that the selection on the population of silenced individuals would yield a homogenous OFF population (comprising those that successfully tip over the potential barrier).

For the second possibility – bistability with noise – the silencing duration *T* shows an extreme (super-exponential) sensitivity to the relative depth of the wells, the height of the barrier, and the magnitude of the noise^40,41^ – as emphasized by the steep dependence of *T* on *V* that we will discuss below. For all *V* ≫ *V_crit_* (strong bistability), we therefore expect to have *T* = ∞. Empirically, while silencing duration is longer than would be expected by dilution, and while such stable silencing is a possible regime of the system (attainable in some mutants), it does not correspond to silencing in wild-type worms (Figure 1C). The system is therefore unlikely to be positioned in the regime of strong bistability.

Other experimental observations are also less compatible with the bistable regime, even when bistability is relatively mild and noise is large. Bistability is characterized by rapid suppression of fluctuations around the stable silenced fixed point, where occasionally large noise allows for escape and de-silencing. On the transgenerational timescale, this results in nearly memoryless transitions between the ON/OFF state. Such memoryless transitions are hard to reconcile with the potency of selection on variation between worms (Figure 1D), as selection on variation in silencing levels in one generation would not be expected to prevent de-silencing in the following generation (Methods). From similar considerations, we do not expect to see uniform de-silencing of sister worms, as de-silencing would be expected to occur rapidly and with a fixed probability, regardless of the initial silencing level (Methods).

**Figure 3:**
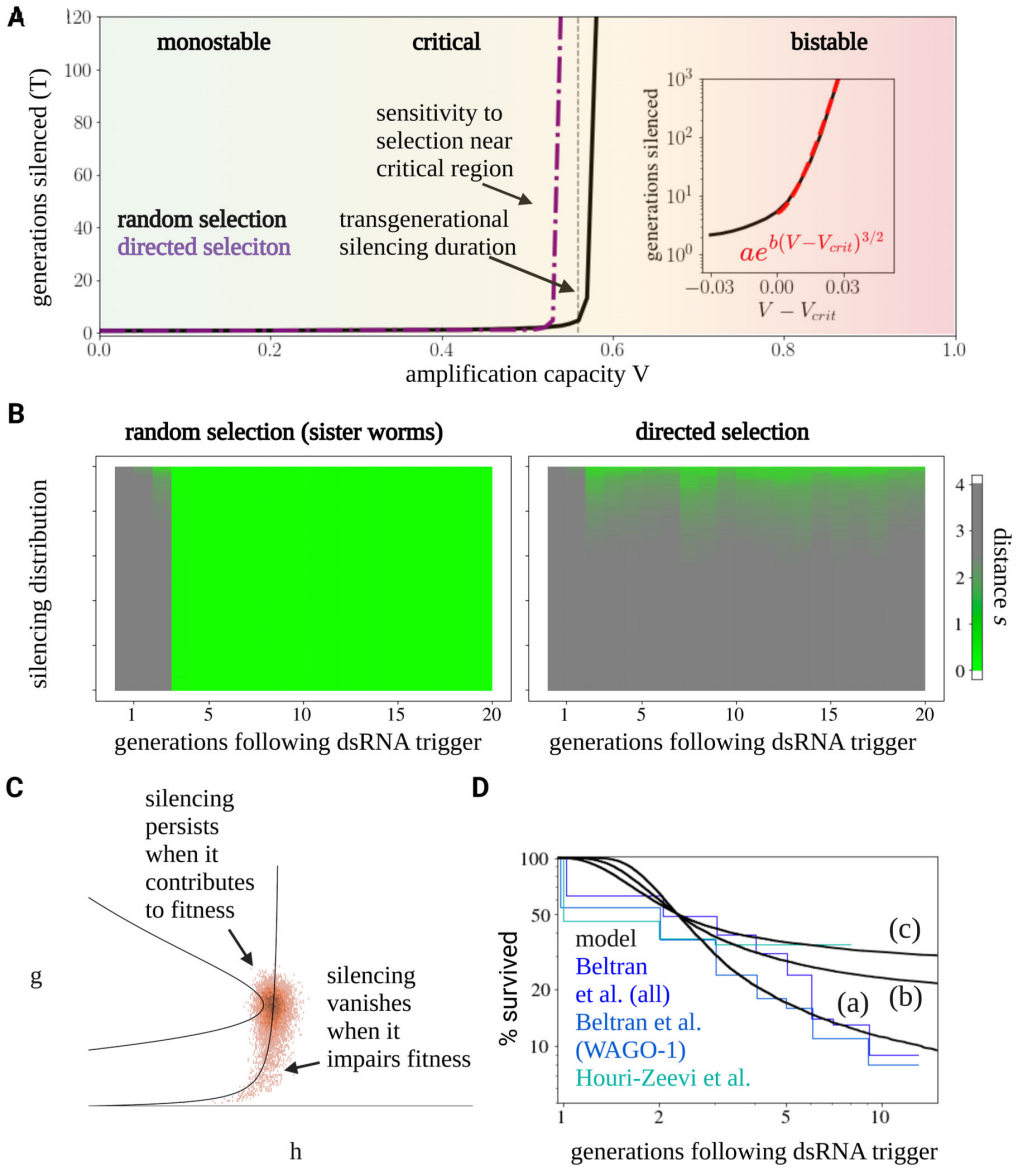
Critical configuration of the TI model provides long term silencing and allows selection on silencing levels. (A) Simulations of the *TI* model were performed under random selection (black) or directed selection of the most silenced individuals (purple) while varying the amplification capacity parameter *V*, exploring the phases of monostability, the critical regime, and bistability. Only around the narrow critical region is there both tunability of the average silencing duration *T*, as well as high sensitivity to selection (as depicted by the large difference between *T* under the conditions of directed vs. random selection). When *V* crosses the critical value *V_CRIT_* (marked by dotted vertical gray line), silencing duration begins to increase rapidly, with a stretched exponential dependence $T\;\propto \;e^{a{\left(V-V_{CRIT}\right)}^{3/2}}$ (*inset*, dashed red line, see main text and Methods for analysis). In the case of directed selection, the silencing degree *s* was assumed operationally to correspond to the Euclidean distance in *g*, *h* phase-space from the unsilenced state at (0,0), since both *g* and *h* may impact on the silencing strength. At each generation, *n* = 250 individual worms were simulated according to the *TI* model dynamics, and the subsequent generation was established by selection either from all worms (*random selection* condition) or by *directed selection* of worms with *s* > *s_select_*, corresponding to strong silencing (here, we took *s_select_* = 3 and set the generation time as 72 hours). (B) Silencing distributions over time in the case of either random selection of a single worm (left panel) or directed selection (right panel) (Methods). Note that, in the panel, all worms in each column are “sister worms”; they de-silence uniformly due to monostable decay away from the critical point. Color scale is provided according to *s*, the (linear) Euclidean distance from (0,0), provided in arbitrary units. (C) The large, static, variation around the critical point allows for selection according to silencing levels. Simulation details are the same as in Figure 2E, where the variation represents the distribution of silencing factors after a single generation starting from above the critical point. All simulations used the same parameters as in Table S1, where *V* is set so the system lies in the critical regime. The critical regime yields a stationary, continuous, distribution of silencing states, as observed by Vastenhouw et al.^20^ (cf. Figure 1D). (D) Variation around the critical point leads to a heavy-tailed distribution of silencing durations due to the high sensitivity of silencing durations around that point. Silencing duration distributions were generated from simulations of the *TI* model with *V* drawn from a normal distribution with mean 〈*V*〉 = 0.53 and (a) 6%, (b) 10%, (c) 15% variation around the mean.

Alongside monostability and bistability, there is a third scenario that is not typically considered in this context– the possibility of a time delay due to a saddle-node remnant (also known as a saddle-node *ghost*)^42^ (Figure 2E), which we have referred to as the critical regime. Saddle-node ghosts are created near a saddle-node bifurcation, such as that found around the transition from a bistable to a monostable regime. In our intuitive picture of monostability and bistability, one may envisage a process (such as changing the amplitude *V*) that raises the metastable OFF (silenced) state to the level of the unstable hilltop. When the two regimes coalesce (the bifurcation), a new and nearly flat slope is formed. A particle that rolls down the slope will be delayed in this region, as if due to a “ghost” of the previous bistable fixed point. This situation occurs when system parameters are fine-tuned, for example by tuning the maximum amplification capacity *V* near a critical value *V_CRIT_*. Near the transition point, the time delay around the critical point, which will dominate the silencing memory time *T,* will have a universal behavior, in the sense that it characterizes all systems undergoing a saddle-node bifurcation near their transition point ^43^. Before the transition, the delay scales in proportion to 𝛥^−1/2^, where 𝛥 ≡ *V_CRIT_* − *V* is the distance from bistability (Methods). Beyond the critical point, where 𝛥 < 0, the delay becomes dominated by fluctuations, with an exponential dependence on 𝛥 (i.e., the delay is proportional to *e*^*b*|Δ|3/2^, where *b* depends on the strength of the noise – for details, see Methods). The delay can therefore become arbitrarily long, and small changes in *V* (translating to small changes in 𝛥) result in large fluctuations in average delay duration (Figure 3A).

In the critical regime, the silencing memory duration, *T*, depends on 𝛥 and, as such, can be effectively decoupled from the turnover rates of the underlying molecular components. Small fluctuations in 𝛥 translate to large differences in silencing times between individuals, characterized by a survival distribution with a nearly flat tail (Methods). When silenced, the silencing state will become “delayed”, and therefore concentrated, near the critical region. Here, the system becomes subject to the phenomenon of *critical slowing down*^44^, where the rate of change of *g* and *h* becomes very small, and the variance (e.g., of the silencing levels) between individual trajectories (worms) increases. Critical slowing down therefore results in large static variation between individuals of the same generation, and the emergence of a long-lasting continuum of silencing states (Figure 2E, lower panel, Figure S3), contrasting the memoryless OFF/ON transitions expected for a mechanism based on bistability. Worms positioned in a state slightly above the critical point are more likely to stay above the critical point during their growth, whereas worms starting below it are likely to lose silencing factors and become de-silenced in the following generation (Methods). This contrasts with the bistable model, where we expect any small initial variation to quickly be eliminated during growth.

The critical regime can provide a credible explanation of the full range of experimental phenomenology described in Figure 1 (as depicted in Figure 3). This regime is compatible with the observed average intermediate silencing duration (Figure 3A). Moreover, it is compatible with entire broad distribution of observed silencing durations, as even small variation in molecular parameters, either between different genes or between different worm lineages, results in silencing distributions with a steep head (viz. silencing closer to the monostable regime, with many worm lineages becoming de-silenced within a few generations) and nearly-flat survival tails (silencing closer to the bistable regime), explaining the observed phenomenology of Figure 1E (Figure 3D).

The phenomenon of critical slowing in the critical regime endows the system with high sensitivity to Selection according to silencing levels (Figure 3A-C, Figure S4, see Movie S3 for animation of a population of stochastic trajectories, and Movie S4 for an animation of deterministic and random selection, Methods). When individuals are selected at random at each generation, progression of the population in phase-space proceeds with a typical delay around the critical region, with a timescale of several generations. By contrast, directed selection of individual worms in which genes are most strongly silenced allows silencing to be maintained for much longer (and potentially indefinite) periods of time: This phenomenon occurs due to the combination of delayed or “static” progression and large variance of the population in the critical region. Recurrent selection of silenced worms leads to the emergence of a persistent distribution of silencing levels in their progenies away from the “de-silenced” state, as observed experimentally (compare the right panel Figure 3B to Figure 1D). Finally, worms that start in states positioned below the critical point progress uniformly towards de-silencing (left panel of Figure 3B), providing an explanation to the uniform de-silencing observed between sister worms (Methods).

It is important to note that none of the above inferences require fine-tuning of model parameters to capture the experimental behavior. Rather, due to separation of timescales compared with dilution, the memory duration is dominated by the delay near the critical point, where properties of interest (such as the time delay as a function of distance from the bifurcation) can be obtained via known scaling behavior ^43^ (Methods).

Taken together, the ghost (critical) mechanism provides a plausible timer mechanism for transgenerational inheritance of silencing in *C. elegans*.

### Robust tuning of transgenerational silencing memory persistence by competition between genes over silencing resources

Despite its success at explaining the observed experimental phenomenology, a major limitation of the ghost mechanism is its apparent reliance on the fine-tuning of the gap 𝛥, the distance of the model parameters from the point of saddle-node bifurcation, to set the silencing duration *T*. Small deviations in 𝛥 lead either to a very short silencing duration *T* (cf. a monostable regime) or very large *T* (a bistable regime), as illustrated in Figure 3A. The mechanism is therefore not robust – noise and variation in circuit parameters can easily change the timescale *T* by orders of magnitude.

At first sight, it is unclear how a biological system can tune and maintain 𝛥 in a robust manner. One possibility is inspired by the concept of self-organized criticality in statistical physics where, under certain conditions, a ghost regime (𝛥 ≈ 0) can emerge as an attractor state ^45,46^, rather than due to fine-tuning of a set of fixed parameters. A classic conceptual example was developed by Bak et al., where a sandpile is formed by slowly adding grains of dry sand^46^. Over time, such a system settles around a critical state, where the addition of new sand grains must, on average, be balanced by the removal of others through avalanches^46,47^. Indeed, the concept of such self-organized criticality has been applied to biological systems in multiple contexts, including genetic networks, brain activity and animal flocks^48–50^, including in particular a classic study on self-tuning to a Hopf bifurcation in the context of hearing^51,52^. More recently, Stanoev et al. have proposed that receptor activity could become self-organized at a saddle-node ghost through fluctuation sensing^53^. However, by itself, self-tuning to criticality (𝛥 ≈ 0) does not present a robust mechanism to tune the timescale *T*, which requires precise tuning of 𝛥 in the vicinity of the critical point.

How, then, is such an apparently fine-tuned regime achieved? In the following, we will show that self-organization of the ghost regime emerges as a consequence of a key feature of the RNA-mediated transcriptional silencing machinery in *C. elegans*, the competition for limited amplification resources (as noted in Figure 1F). Moreover, within the framework of a minimal competition model, we will see that the average inheritance timescale, *T*, can be set robustly, even in the presence of noise and variation between genes.

To account for competition between siRNAs, we developed a straightforward generalization of the *TI* model, which we termed the *Toggle-Inhibitor Competition (TIC)* model. While competition has been considered previously in mathematical models of transgenerational inheritance^17^, these theories, which only account for competition between two silencing pathways, cannot explain the observed tuning. Here, we show that a “many-particle” theory, that accounts for competition between many silenced genes, provides a robust mechanism of fine-tuning. Let us denote by indices *i* = 1, *I*, *N* the ensemble of endogenous and exogenous genes that can become transgenerationally silenced. Silencing events occur stochastically, following from either chance encounters with exogenous challenges such as viruses, or endogenous stochastic silencing events ^10^. Within the framework of the TIC model, we propose a model of silencing dynamics similar to that of the *TI* model but with a global “cost” term *C*(*g*_1_, *h*_1_, *g*_2_, *h*_2_, . .), which inhibits the amplification rate of siRNAs by modulating *V* or *k*_1_ (see Movie S5 for the effects of modulation *V* or *k*_1_ on the nullclines). Here, for simplicity, we begin by supposing that the model parameters, *k*_1_, *k*_2_, etc. do not vary between genes. To demonstrate the role of resource competition in the TIC model, we will consider a simple scenario in which the cost function modulates the amplitude $\text{V}=\frac{{\text{v}}_{\text{tot}}}{c\left(\left\{g_{j},h_{j}\right\}\right)}$ with *C* = Σ*_j_g_j_*, noting that other possibilities, including modulation of *k*_1_ or other choices of *C* lead to similar dynamics (Methods). In this case, the TIC model translates to the kinetics

(3) $$dg_{i}=\left(I_{i}\left(t\right)+\frac{{\text{V}}_{\text{tot}}}{\sum_{j}g_{j}}\frac{g_{i}{}^{n}}{k_{1}{}^{n}+g_{i}{}^{n}}\frac{k_{2}}{k_{2}+h_{i}}-\gamma_{1}g_{i}\right)dt+S_{1}\left(g,h\right)dW_{1}$$

(4) $$dh_{i}=\left(\psi \frac{g_{i}}{k_{3}+g_{i}}-\gamma_{2}h_{i}\right)dt+S_{2}\left(g,h\right)dW_{2}$$

where, in this case, *I*_*i*_(*t*) is a stochastic random variable. For example, I_i_(*t*) may acquire positive values at a constant rate, so that the number of silencing events per generation is characterized by a Poisson distribution. Note that we have also replaced the amplitude *V* by the parameter V_tot_, as this now denotes the amplification capacity that is shared between genes.

**Figure 4:**
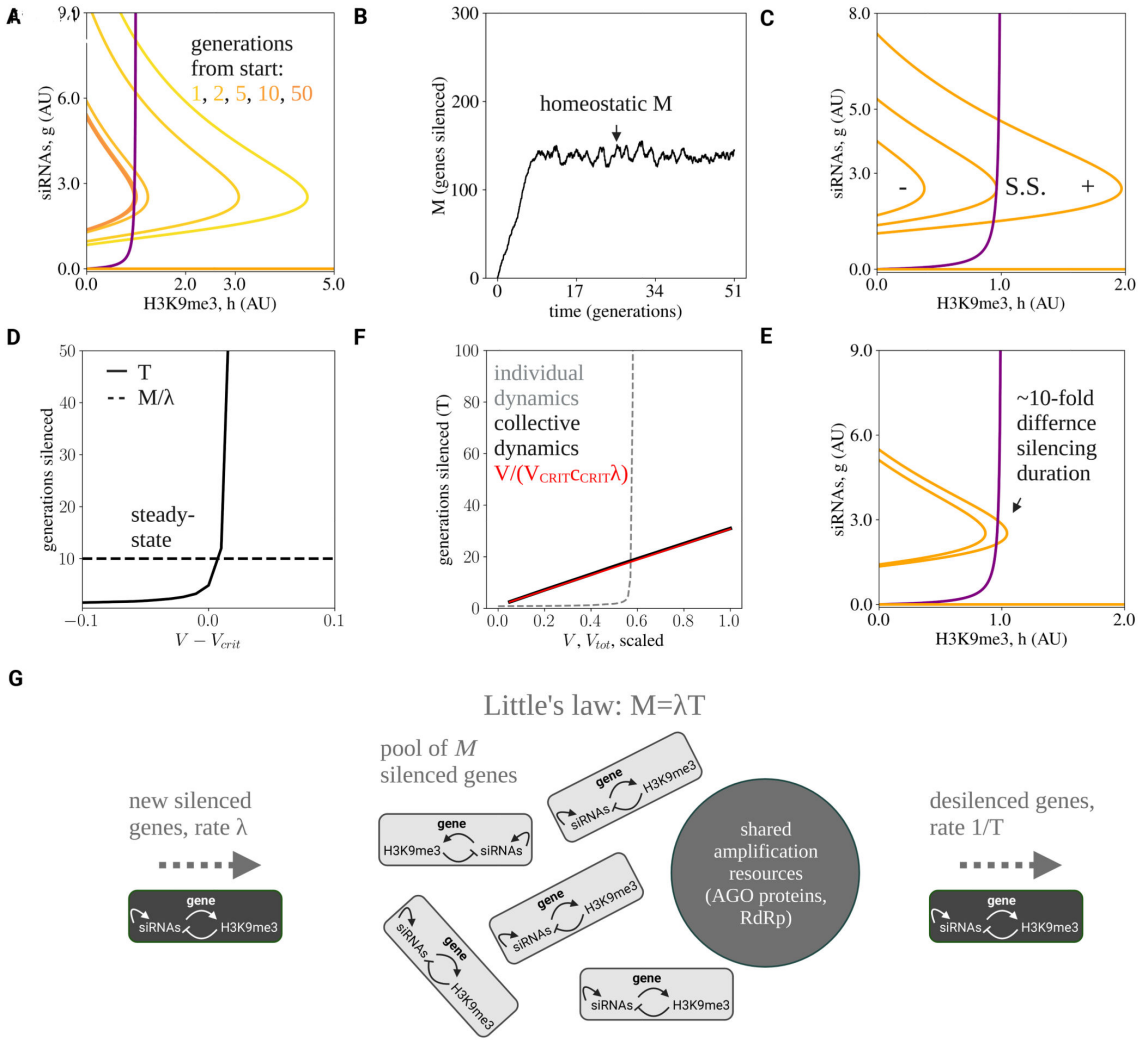
Robust self-organization of long-term gene silencing driven by competition for silencing machinery. In the *Toggle-Inhibitor-Competition (TIC)* model, stochastic events in a pool of candidate genes drives silencing transitions. We denote by *M* the average number of silenced genes at steady-state, and by λ the average rate at which new silencing transitions occur stochastically. (A) Starting with an initial condition in which all genes are unsilenced, stochastic simulation of the *TIC* model shows nullclines $\dot{h}=0$ (purple) and $\dot{g}=0$ (orange, different lines depict generation number) settling near the region of saddle-node bifurcation over time. Note the rapid convergence from bistability towards the critical regime between bistability and monostability. (B) Over time, the system settles into a stable homeostatic phase in which, at any given time, an average of *M* genes are silenced. (C) The configuration of nullclines $\dot{h}=0$ (purple) and $\dot{g}=0$ (orange, different lines for monostable, ghost and bistable configurations) set by the bifurcation ratio of the amplitude and cost function *V*_tot_/*C*, determines the average silencing duration time, *T*. When the system is in the bistable regime (right-most orange nullcline), silenced genes accumulate (+); while in the monostable regime (left-most orange nullcline), they are removed (-). There is thus feedback from the size of the silenced gene pool *M* back to the bifurcation parameter through the cost term driving the system to steady-state (S.S.). (D,E) Schematic illustration of the mechanism underlying the tuning of *T*. According to Little’s law (depicted graphically in panel E), at steady-state, *M* = *λT*. Note that, while the silencing duration *T* (panel D) changes rapidly around the bifurcation point (results from stochastic simulation as in Figure 3A), *M* is much more narrowly distributed (panel B), fixed by the cost function near the bifurcation. This narrow range of *M* effectively sets the average duration time *T* = *M*/*λ*. (F) The silencing duration *T* is proportional to *V*_tot_ (black line, results of stochastic simulation while varying *V*_tot_ between 50-1000) and can be recovered by a simple analytical formula (red line, see main text and Methods). This behavior contrasts with the high (exponential) sensitivity of silencing duration to the amplification parameter *V* in the *TI* model (gray line, stochastic simulation of *TI* model while varying *V*). (G) The linear dependence corresponds to small changes in the positions of the nullclines around the critical point (shown here are the effective nullclines when *V*_tot_ = 100 or *V*_tot_ = 1000, corresponding to a ~10-fold difference in *T*). Simulation parameters are provided in Table S2, and simulation details and code are provided in Methods. While the simulations did not assume variation in circuit parameters between genes, similar results are obtained when this variation is incorporated (Methods).

For simple circuits consisting of linear production and removal, competition for synthesis resource would reduce the overall activity level, leading to variation in the steady-state or amplitude without affecting response duration. However, for the *TIC* model, a distinct and different dynamical pattern emerges (Figure 4). Consider a situation in which the system begins in a state with no silenced genes, so that the initial cost function *C* is small and the amplification capacity large, placing the system in a phase of bistability. Under these conditions, silenced genes will begin to accumulate through stochastic silencing events, leading in turn to a gradual increase in the cost function *C*. Stochastic simulations of the model dynamics are depicted in Figure 4A,B, as well as Movie S6 in animation. If this increase occurs rapidly, the cost function may increase to a level that places the system in a monostable regime, where genes now become rapidly de-silenced, leading in turn to a decrease in *C*. Based on these dynamics, this process will continue until the system eventually stabilizes close to the region of bifurcation between bistability and monostability, where the ghost regime is defined (Figure 4B). Of course, in practice, convergence to this phase from the bistable regime may not be characterized by such a damped oscillatory dynamic, but the approach may be monotonic. Yet, within this framework, the stability of the critical ghost regime in the long-term is assured with no requirement for fine-tuning of biochemical parameters.

To understand why the system must settle near the saddle-node bifurcation, it is instructive to consider the dynamics of the system from the perspective of stochastic queuing theory (Figure 4C-E). Here, the siRNA silencing machinery can be considered as a “server” and silenced genes as “clients” that arrive at random and are handled by the server before becoming de-silenced. If *M* denotes the average number of genes that are silenced when the system is in steady-state, from the intuition above, it follows that the system can be bistable at steady-state only when *M* = *N*, the total number of genes that can be silenced. This situation will prevail only when the amplitude V_tot_ is sufficiently large. However, at smaller values of V_tot_, *M* ≪ *N*, and the system must transition to the critical phase of monostability long before all genes become silenced. In this case, if we denote by *λ* the average arrival rate of new silencing events (a property of the stochastic input rate *I_i_*(*t*)), at steady-state the average silencing duration would be given by *T* = *M*/*λ*, a general property that follows from Little’s law for the steady-state dependency of a queuing system ^54^. As the system transitions stochastically between bistability to monostability, *M* varies around a narrow range (due to small changes in the cost parameter *C*), while silencing durations of individual genes *T* cover a wide range of possible values. The system thus fixes the average gene silencing duration *T* according to the relation *T* = *M*/*λ* in the critical region. We note that, even in the presence of noise, which has the effect of pushing the system marginally towards bistability, the system will settle near the critical point due to the very steep (exponential) dependence of *T* on the amplitude *V* (as depicted in Figure 4D, see Methods). We thus term the mechanism *self-tuned criticality by competition.*

The *TIC* model provides a robust mechanism for tuning the average silencing duration *T* independently from the individual turnover rates of the underlying molecular components. The capacity *M*, the average number of silenced genes at any given time, can be derived directly from the model – it is fixed by the average cost per silenced gene near the bifurcation point *c*_CRIT_, and is proportional to the maximal amplification capacity, *V*_tot_/*c*_CRIT_ (Methods; note that, here, *c*_CRIT_ is simply *g*_CRIT_, the value of *g* at the critical point). This holds even when considering noise or statistical variation in circuit parameters between genes (Methods). We can therefore think of the *TIC* model as defining a mechanism that translates the amplification capacity *V*_tot_ into an average silencing memory time *T* (Figure 4E,F).

In the competition model, there is a gradual (linear) dependence of *T* on *V*_tot_ and other circuit parameters, which contrasts with the steep dependence of *T* on *V* for individual memory dynamics near the critical point (Figure 4E,F,G). The linear dependence of the average memory time on *V*_tot_ allows for robust tuning of silencing duration since changes in *V*_tot_ only lead to proportional changes in *T*. This allows for regulatory programs and evolutionary pressures to robustly tune and adjust a “forgetting time” on the order of many generations.

The change in *T* following a change in circuit parameters is due to the collective dynamics of the silencing memories. For example, a change in *V*_tot_ changes the size of the steady-state silenced gene pool *M* (*M* → *M*′). The dynamics of the change may be asymmetric in their dependences: A downwards change in *M* shifts transiently the system into the monostable regime, leading to rapid de-silencing. On the other hand, depending on the scale of *λ*, an upwards change in *M* increases the number of silenced genes over a time scale on the order of *T* = *M*′/*λ* (Methods). Similar dynamics also hold for an individual silenced gene, which upon a downwards change in *M* may become de-silenced rapidly; but upon an upwards change, will become re-silenced within a typical timescale of *N*/*λ* > *T*, under the minimal assumption that all genes are silenced at the same rate.

### Self-tuned criticality by competition in the TIC model is consistent with established experimental properties of the epigenetic inheritance system

The proposed competition mechanism has several important quantitative features that are consistent with the known experimental phenomenology of the transgenerational inheritance system. The foremost is the association between amplification capacity and silencing duration, described in Figure 1F. For example, in the mutants depicted in Figure 1F, the extremely long silencing duration (over 300 generations) can be readily understood within the framework of the TIC model because of the freeing up of amplification capacity *V* following loss of endogenous small RNAs. Similarly, this mechanism can also explain the long-term silencing in *met-2* mutants, which also have a strong downregulation of endogenous small RNAs^55^. More generally, the model is consistent with the established associations between competition between genes over silencing resources, RNAi response sensitivity, and silencing duration^17,27–32^.

The timescales of gene dynamics are also consistent with experimental observations on the system. The slow recovery from a downwards to upwards change is evident in an experimental study by Klosin et al.^14^. Animals expressing a stochastically-silenced multi-copy reporter transgene were raised in high temperature conditions (25 °C), which causes the de-silencing of endogenous genes (viz. a step change in some physiological parameters). Re-silencing can be established by transferring the worms back to 20 °C (reversing the previous step change). Klosin et al. demonstrated that a mere 48-hour incubation in 25 °C caused a de-silencing that persisted for 7 generations, while a 5-generation incubation in 25 °C caused de-silencing that persisted for 14 generations. In the model, the slow dynamics of recovery reflect the stochastic nature of the accumulation process, with a re-accumulation timescale that is longer than the memory duration *T* (as anticipated by the model), while the loss dynamics may be much faster (corresponding to a transition of the system to the monostable regime).

### The TIC model makes specific predictions that are amenable to experimental validation

The TIC model proposes that the transgenerational epigenetic silencing system is self-tuned to the vicinity of a saddle-node bifurcation because of competition over shared silencing machinery between silencing memories. Both aspects make distinct predictions that can be tested experimentally.

**Figure 5:**
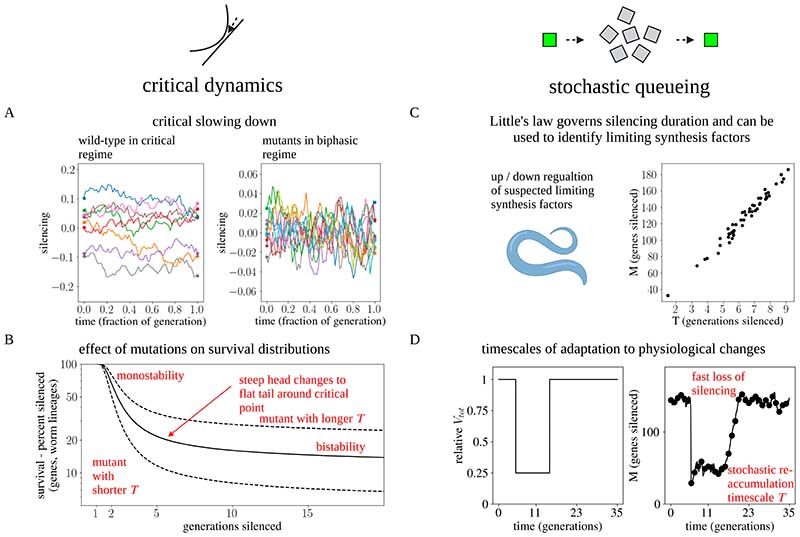
Predictions of TIC model. (A) The TIC model predicts that the dynamics of the silencing state are subject to the phenomena of critical slowing down, which results in correlated silencing levels throughout the growth process. The model therefore predicts that, amongst silenced worms, those that have slightly below average silencing in early life are more likely to become de-silenced either later in life or in the following generation, while those that have above-average silencing are more likely to stay silenced throughout life and in the next generation. This contrasts with worms in the bistable regime (such as worms lacking piRNAs), where we predict that any variation should become rapidly suppressed during the growth process. This may be measured by following *gfp-*silenced worms longitudinally over several days. (B) Survival distributions of genes and worm lineages when the silencing system is placed at the critical point have a characteristic shape (solid line) with a steep head (corresponding to instances closer to the monostable regime) and a heavy tail (corresponding to instances closer to the bistable regime), with an “elbow” around the silencing duration at the critical point. The model predicts that, for mutants where silencing duration is modulated through altered synthesis capacity, the “elbow” region should stay at the same silencing duration, but the fraction of instances closer to monostability/bistability should become modulated (dashed lines). (C) Modulation of limiting synthesis factors would result in correlated changes in the size of the silenced gene pool *M* and silencing duration *T*. (D) The model predicts that changes in *M* have asymmetric transient timescales – down changes in *M* may be rapid due to transition to the monostable regime, while up-changes are slower as they depend on stochastic silencing events.

To test whether the system is tuned to the vicinity of a saddle-node bifurcation, one can exploit the singular properties that characterize this state, known in the parlance of critical phenomena as critical slowing down^44^. Generic properties of critical slowing down are due to the slow decay of fluctuations around the critical point and result in phenomena such as a slow decay of temporal autocorrelation. More specifically, we expect that, in the generations following the silencing of a gene by a dsRNA trigger, worms with a stronger silencing (corresponding to silencing state above the critical point) would mostly remain silenced throughout their life and in the following generation, while worms with weaker silencing (corresponding to silencing below the critical point) would transition towards de-silencing (Figure 5A). This prediction underlies the model’s explanation regarding the high efficacy of selection. It may be possible to quantify this temporal autocorrelation experimentally by measuring fluctuations in *gfp* expression in individual worm lineages following dsRNA treatment over the timescale of days. The high autocorrelation predicted for wild-type worms should contrast with the much lower autocorrelation times predicted for worms in the bistable regime, such as worms devoid of piRNAs^19^.

Another way to test whether the system is positioned near the saddle-node bifurcation point is by exploiting scaling properties around that point. These properties manifest in the distribution of *T* for different genes or different worm lineages. Due to the steep dependence of *T* around the critical point, the survival distribution of silencing memories is characterized by a steep head and a nearly flat tail, with the transition between the two regimes corresponding to the delay around the critical point itself (Figure 5B). Moreover, assuming a Gaussian distribution of model parameters around the critical point, the distribution of silencing durations itself only depends on the (scaled) variance and the offset from the critical point (Methods). It is therefore possible to test whether the empirical survival curves of *T* under different conditions (temperature, mutants, etc.) indeed correspond to the predicted distributions.

The other aspect that can be tested is the tuning process, which is predicted to be due to competition over limiting synthesis factors. It is currently unclear what may be the main limiting factors, but possible candidates include the abundance of RdRp molecules and AGO proteins. The limiting factor(s) may be identified experimentally according to the model predictions. The model predicts that manipulating the limiting factor should coordinately increase both the size of the steady-state silenced gene pool *M* and the silencing duration *T* (Figure 5C). The model predicts a gradual dependence of *M* and *T* on the abundance of the limiting factor. It also predicts that a down-shift of the limiting factor will result in a rapid reduction in *M* due to the transition to the monostable regime, while an up-shift will increase *M* on the timescale of *M*/*λ* = *T* (Figure 5D) due to stochastic silencing events. These predictions can be tested by performing genetic manipulations on suspected factors, together with quantitative measurements of the silenced gene pool through small RNA-seq and ChiP-seq. Finally, it will be interesting to test whether a large up- or down-manipulation of synthesis factors can transition the system to bistability or monostability, where the hallmarks of critical dynamics are predicted to be lost.

## Discussion

Epigenetic gene-silencing in *C. elegans* identifies a general design principle for the implementation of long-term memory by biological circuits. We showed that the wide range of experimental phenomenology may be captured by a response system positioned close to a saddle-node bifurcation. By combining a mathematical modelling-based approach with transcriptional and chromatin measurements, we found that the dynamics of gene silencing are consistent with an excitable circuit model, where silencing is driven by a bistable switch, followed by inhibition. Competition between silencing memories for limited resources self-tunes the response system near to a saddle-node bifurcation. These core interactions, encapsulated by the *Toggle-Inhibitor-Competition* model, provide a robust timer mechanism for transgenerational silencing dynamics.

The *TIC* model contrasts with classical models of biological memory. From the perspective of biological circuits, long-term memory is often attributed to bistable switches, which can transition between stable ON/OFF states^39,56,57^. Bistable switches arise in systems with nonlinear positive feedback^58,59^. An activating stimulus can shift the system from the OFF to the ON state, and the system will remain in the ON state even after the stimulus has been removed. Bistable switching is thought to stand at the core of a myriad of biological processes, including the control of cell cycle transitions^60,61^, cellular differentiation^62^, vernalization^63^, and immune cell homeostasis^64^. By contrast, in the *TIC* model, although activation dynamics are governed by bistability, the system becomes self-tuned to the vicinity of a critical regime, and the delay in this regime determines memory lifetime.

While bistable switches allow biological systems to maintain memory, they have several drawbacks that hinder their applicability in memory systems such as epigenetic silencing. The first issue concerns the maintenance of a robust “forgetting time” (or survival time) *T* that is neither too short nor too long. Forgetting requires spontaneous transitions between states in the absence of an explicit trigger, leading to a typical exponential dependence on both the magnitude of noise and circuit parameters (as exemplified by the escape rate from a potential well for gradient systems, but which can be extended to non-gradient systems via large deviations theory)^40,41^. Such exponential dependence is, however, not robust as it corresponds to a high sensitivity of *T* to specific cellular conditions.

The second issue concerns the ability to retain certain relevant memories over other memories, according to inputs to the system. This is exemplified in the transgenerational silencing system by the sensitivity to selection on silencing levels – the critical regime endows the system with a continuum of nearly static silencing levels, which allows for efficient selection. This contrasts with the monostable regime, where trajectories advance in phase-space with each generation, and the bistable regime, where fluctuations decay rapidly towards steady-state. In the critical regime, selection of worms with stronger silencing provides a perturbation that prevents average silencing from decaying away from the critical point. More generally, one can consider other perturbations that move the memory state in phase space, including continuous input signals. As an example, in the epigenetic memory system, repeated priming resulted in extension of silencing retention^17^. Self-organization near the critical point provides a mechanism for integrating these perturbations in order decide on memory retention^53^.

The TIC mechanism decouples the average memory duration *T* from the underlying molecular dynamics. According to the model, *T* is given by the ratio of the size of the memory pool *M* (fixed by the location of the critical point in phase-space) to the arrival rate of gene silencing events λ. Indeed, it is also possible to decouple *T* from λ by adding a feed-forward activation of synthesis resources *V* by memory arrivals, as this would normalize away λ from *T* (Methods).

While self-tuning is a robust model property, it may be broken under certain circumstances, which may constitute “weak points” of the system. Tuning to the critical point holds as long as the size of the silenced gene pool, *M*, is much smaller than the size of the pool of genes that can become silenced, *N*. If *M* approaches *N*, however, we expect that new silencing events would become rarer, and *T* would rapidly increase, becoming effectively infinite at *M=N*. On the other hand, changes in parameters that result in lower *T*(for example, an increase in the stochastic arrival rate λ) may transition the system to the monostable regime where selection becomes ineffective. This presents a vulnerability, as transposable elements that replicate and mutate in the genome may increase λ and therefore effectively de-silence themselves. This can result in an arms race-like dynamics between transposable elements and the overall synthesis capacity, which may explain why piRNAs (which are associated with silencing these elements) have such large impact on the available silencing machinery.

In this study, we proposed a simple realization of the *TIC* model based on autocatalysis of target-specific siRNAs and negative feedback from silencing chromatin marks. While such a model is plausible based on the known biological interactions and dynamical measurements, there are likely to be other important regulatory interactions that affect transgenerational inheritance dynamics. However, due to the generality of the model assumptions, as well as scaling behavior near the critical point, the important quantitative aspects of the model are preserved even in more complex and higher-dimensional settings. For example, one may show that the nature of the dynamics is unchanged when including into the dynamical equations pUG RNA-templates from which siRNAs are synthesized (^65^, Methods). Moreover, the observation that competition over synthesis resources leads to self-organization near a critical point allows us to make quantitative predictions on the properties of the system (such as the distribution of memory durations) despite having only limited knowledge of the underlying regulatory network.

**Figure 6:**
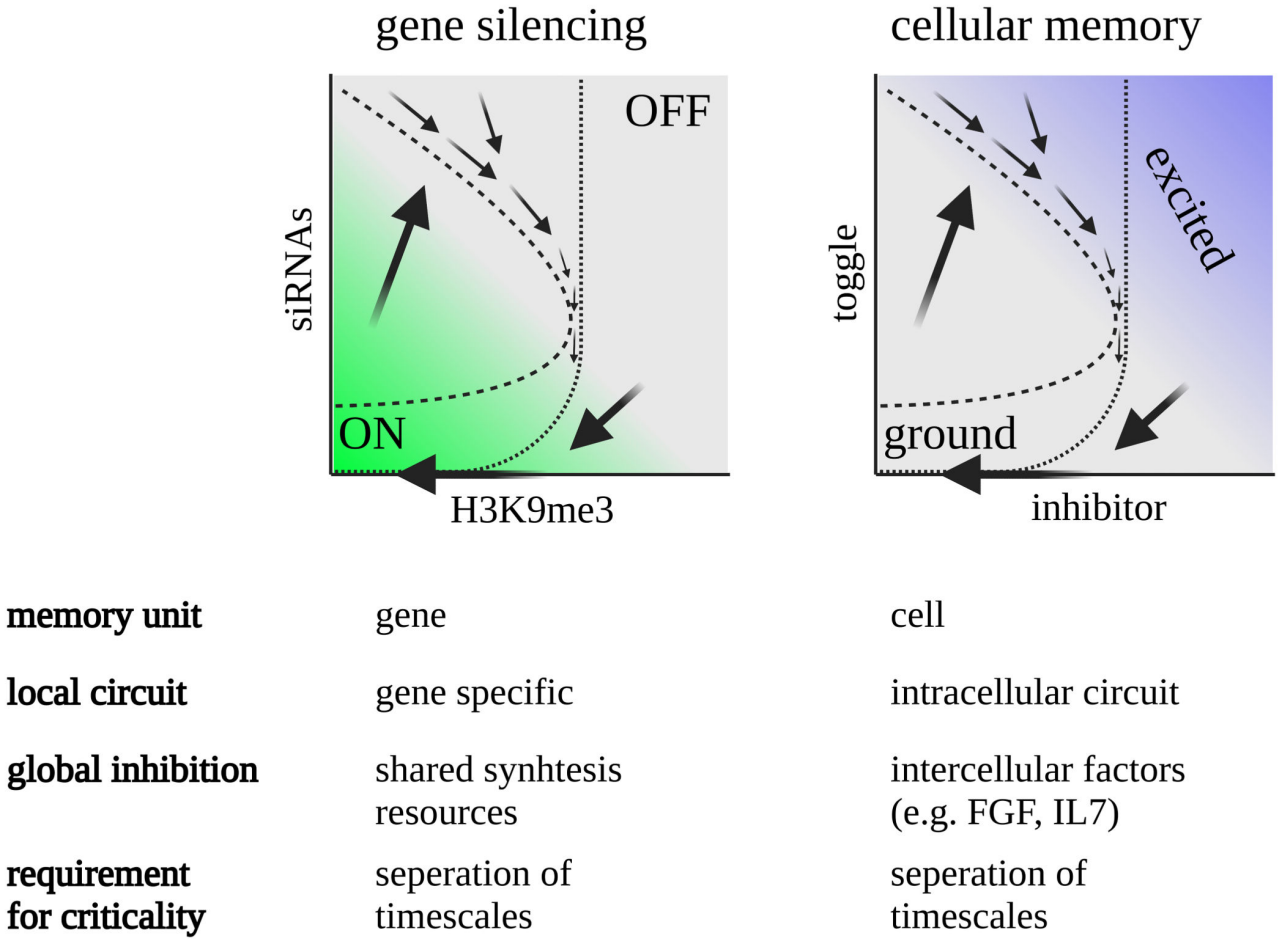
Generalization of self-tuned criticality by competition to cell-based memory. The *TIC* model can be generalized to cell-based memory circuits, where the “local” memory unit is a cell (compared with a gene for the silencing mechanism in *C. elegans*). The model requires an intracellular excitable circuit separating “ground” and “excited” states, similar to the excitable high/low Nanog circuit described by Kalmar et al. in the context of embryonic stem cells ^66^. In the immune system, the excited state may correspond to a memory identity associated with a particular antigen. It also requires global feedback that can transition the system between monostable and bistable states, which may be implemented by known quorum sensing mechanisms. Finally, self-organization near the critical state is achieved when there is a separation of timescales between the steady-state memory duration and the turnover rates of the underlying molecular circuit.

Given the simplicity of the model, it makes sense to question whether the concept of self-tuned criticality by competition can be extended to other memory systems, including cell-based memory systems such as the mammalian immune system, as well as cellular interaction networks (Figure 6). We, therefore, summarize the few necessary ingredients that such a memory system should possess, and the signature properties that may indicate that such a mechanism is at play. Specifically, for the mechanism to operate, the system needs to have memory response dynamics governed by an excitable circuit, with cooperative activation and negative feedback that are memory-specific. In the transgenerational silencing system, these memory-specific interactions may arise by siRNAs that silence the same target sequence. In cellular systems, these may correspond to intracellular excitable circuits; indeed, such circuits have been implicated in the process of cellular differentiation^66,67^. In addition to the memory-specific excitable circuit, the model also requires *global inhibition*, which can act as a control parameter for the transition from monostability to bistability in individual memories. Global inhibition can occur in different flavors. However, the simplest form is inhibition of the amplification rate through competition over synthesis resources. In the case of the *C. elegans* inheritance system, there is competition of AGO proteins and RdRP complexes. In the immune system, T-cells compete over global factors such *IL-7* that control overall T-cell levels^68^, while other factors such as *FGF* play an important role in mediating quorum feedback in cellular dynamics^69,70^. Such mechanisms are widely regarded in the biological literature as *homeostatic*, as they maintain the overall levels of memory units within a fairly narrow range. Finally, it is required that the average memory timescale *T* be longer than the typical timescale of the turnover rate of the excitable memory circuit. The combination of fast excitable memory-specific dynamics with global inhibition keeps the system near bifurcation by pushing it away from bistability and allows for robust tuning of memory duration *T*.

The model makes several predictions that can serve as a signature when considering its potential applicability in other memory systems. First, in the framework of TIC dynamics, memory duration depends on collective memory dynamics. In an “open loop” setting, where collective feedback is not active, memory duration is predicted to show very high sensitivity to circuit parameters around the critical point while, in the “closed loop” setting, self-organization around the critical point leads to only a mild dependence of *T* on circuit parameters. Second, according to the model, memory duration may be adjusted by changing the memory pool size by adjusting synthesis capacity, or by modulating the arrival rate *λ*. Moreover, the average memory duration *T* can be inferred by examining the time until the re-establishment of the memory repertoire from stochastic arrival events. Finally, the model predicts a distinct distribution of memory survival times that results from its convergence at steady-state towards the vicinity of a saddle-node bifurcation (Methods), with a relatively steep head corresponding to events taking place closer to the monostable regime, and a flat tail (that asymptotes towards a nearly flat dependence on *T*) corresponding to events taking place closer to the bistable regime.

While the transgenerational silencing of endogenous and exogenous genes in *C. elegans* is well established, the evolutionary benefits of the mechanism remain a topic of intensive research, with a focus on adaptation to fluctuating environments and for pathogen memory^15^. A particularly intriguing aspect is the widespread stochastic silencing of genes^10^. Stochastic, transient silencing provides a mechanism for stochastic phenotypic switching, which can have several functional benefits such as bet-hedging against environmental uncertainty^71–74^. Our model provides a generic mechanism for the implementation of phenotypic switching over intermediate timescales. Moreover, rather than binary switching between discrete states, our model suggests that silencing at the population level can be maintained at a heritable continuum. Thus, if a rare stochastic silencing event provides an unexpected benefit (such that stronger silencing is associated with higher fitness), it can be maintained for a longer duration, while detrimental silencing events are discarded. This may allow worms to adjust and adapt their pattern of gene expression in response to changing environmental conditions.

In conclusion, competition in the TIC model provides a robust mechanism for tuning long-term memory persistence and presents features beneficial for the retainment of relevant memories.

## STAR Methods

### Resource Availability

#### Lead Contact

Further information and requests should be directed to and will be fulfilled by the lead contact, Omer Karin.

#### Materials Availability

The study did not generate any new materials.

### Experimental Model and Subject Details

The study did not use experimental models.

### Method Details

#### Toggle-Inhibitor (TI) model

In the following sections, we provide further details on the Toggle-Inhibitor (TI) model of gene silencing and its generalization, the Toggle-Inhibitor-Competition (TIC) model. Specifically, we use analytical approaches to determine the properties of these models including their phase behavior, stability, dynamics, and the effects of noise, as well as presenting details of the stochastic simulations used to model the different conditions considered in experiment.

The TI model is based on excitable dynamics with an effector *g* (the concentration of gene-specific 22G siRNAs) and an inhibitor *h*(the concentration of silencing marks H3K9me3 on the target gene). In the absence of noise, its dynamics are given by the coupled set of rate equations:

(5) $$\dot{g}=I\left(t\right)+V\frac{g^{n}}{k_{1}^{n}+g^{n}}\frac{k_{2}}{k_{2}+h}-\gamma_{1}g$$

(6) $$\dot{h}=\psi \frac{g}{k_{3}+g}-\gamma_{2}h$$

where *I*(*t*) denotes the source term or trigger. Since both *g* and *h* contribute to silencing, we can consider the degree of silencing *s* to be an increasing function of *g* and *h*, such as the Euclidean distance from the origin: $s=\sqrt{g^{2}+h^{2}}$. The effector is catalyzed through cooperative autocatalysis with a Hill-coefficient *n* > 1, maximal synthesis rate *V*, and half-way saturation *k*_1_. Synthesis is inhibited by *h*, with a half-way inhibition *k*_2_. There is also a production term (e.g., due to dsRNA triggers) set by *I*(*t*). The inhibitor is synthesized by *g* with half-way saturation *k*_3_ and maximal amplification rate *ψ*. The effector *g* decays with rate *γ*_1_ and the inhibitor *h* decays with rate *γ*_2_. For simplicity, we consider all the concentration variables *k*_1_,*k*_2_,*k*_3_ as defined in arbitrary units of concentration (denoted AU).

The TI model captures the dynamics of silencing factors on a multi-generational timescale, and therefore incorporates the effect of dilution due to growth and reproduction. We denote by *τ* the time scale of a single generation, which is on the order of days (see Table S3). Time scales can thus be divided into generations, starting from *t* = 0 (first generation, second generation, etc.). For the rate parameters, we note that the dynamics on the transgenerational timescale are dominated by dilution, and a reasonable estimate for the decay rates *γ*_1_, *γ*_2_ would be on the order of hours (see main text).

#### TI model nullclines

To understand the dynamics of Eqs. (5,6), we first consider their nullclines, where $\dot{g}=0$ or $\dot{h}=0$. For simplicity, we will start by setting *I*(*t*) = 0, noting that a non-zero production rate changes the nullclines (a situation that we will analyze later). The nullclines for Eq. (5) are given by:

(7) g=0

(8) $$h=k_{2}\left(\frac{V}{\gamma_{1}}\frac{g^{n-1}}{\left(g^{n}+k_{1}^{n}\right)}-1\right)$$

while, for Eq. (6):

(9) $$h=\frac{\psi }{\gamma_{2}}\frac{g}{k_{3}+g}$$

The nullclines are illustrated graphically in Figure S5 for sample parameters. The nullcline of $\dot{h}$ is a Michaelis-Menten curve, which saturates at a maximum *h* = *ψ*/*γ*_2_, while the non-trivial nullcline of $\dot{g}$ is a unimodal curve with an extremum at position *p*_max_ = (*g*_max_,*h*_max_) where, solving *∂h*/*∂g*= 0 on the nullcline, one finds that:

(10) $$\begin{array}{cc} g_{\max}={\left(n-1\right)}^{1/n}k_{1}\approx k_{1}, & h_{\max}\approx \left(\frac{V}{2\gamma_{1}k_{1}}-1\right)k_{2} \end{array}$$

Thus, for *V*/*γ*_1_ < 2*k*_1_, the non-trivial nullcline takes values only at negative *h*, and only the trivial nullcline is relevant. Also note that, for *g* ≫ *k*_1_, the nullcline decays approximately linearly as $h\approx k_{2}\left(\frac{v}{\gamma_{1}g}-1\right)$.

Another way to visualize this is by considering the nullclines Eqs. (8,9) as surfaces that depend on the parameters. As can be seen in the illustration Figure S6, there is a specific point in the g-h-V parameter space corresponding to the critical point where the nullclines intersect only once over the g-h cross section.

#### Fixed points and stability

The fixed points of the system are given by the intersections of the nullclines of Eqs. (5,6). For the trivial nullcline *g* = 0 there is one intersection at *p*_1_ = (0,0). To assess the stability of this fixed point, we can use linear stability analysis, forming the Jacobian,

(11) $$\text{J}=\left[\begin{array}{ll} \partial_{g}\dot{g} & \partial_{h}\dot{g}\\ \partial_{g}\dot{h} & \partial_{h}\dot{h} \end{array}\right]=\left[\begin{array}{cc} V\frac{ng^{n-1}k_{1}^{n}}{\left(k_{1}^{n}+g^{n}\right)}\frac{k_{2}}{k_{2}+h}-\gamma_{1} & -V\frac{g^{n}}{k_{1}^{n}+g^{n}}\frac{k_{2}}{{\left(k_{2}+h\right)}^{2}}\\ \psi \frac{k_{3}}{{\left(k_{3}+g\right)}^{2}} & -\gamma_{2} \end{array}\right]$$

At the fixed point *p*_1_, the Jacobian:

(12) $$\text{J}\left(p_{1}\right)=\left[\begin{array}{ll} -\gamma_{1} & 0\\ \frac{\psi }{k_{3}} & -\gamma_{2} \end{array}\right]$$

has two negative eigenvalues *l*_1_ = −*γ*_1_, *l*_2_ = −*γ*_2_, and is therefore stable, with decay of the state towards *p*_1_ dominated by dilution.

The existence of other fixed points depends on the location of *p*_max_ and whether the non-trivial nullcline of $\dot{g}$ intersects the nullcline of $\dot{h}$. There may be zero, one, or two intersection points. Here, we will analyze the case where the synthesis rate is large, so there are two intersection points (see Figure S5). Taking *g* ≫ *k*_1_, the first intersection point, which we denote as $\left(g_{p_{2}},h_{p_{2}}\right)$, occurs at $g_{p_{2}}\approx \frac{\text{v}}{\gamma_{1}\left(\frac{\psi }{\gamma_{2}k_{2}}+1\right)}$ with $h_{p_{2}}$ obtained from Eq. (9). Then, noting that

$$\begin{array}{c} V\frac{ng^{n-1}k_{1}^{n}}{{\left(g^{n}+k_{1}^{n}\right)}^{2}}\frac{k_{2}}{k_{2}+h_{p_{2}}}\approx \frac{k_{2}nVg^{-n-1}k_{1}^{n}}{h_{p_{2}}+k_{2}}\to 0\\ -\frac{k_{2}Vg^{n}}{{\left(h_{p_{2}}+k_{2}\right)}^{2}\left(g^{n}+k_{1}^{n}\right)}\approx -V\frac{g^{n}}{k_{1}^{n}+g^{n}}\frac{k_{2}}{{\left(k_{2}+h_{p_{2}}\right)}^{2}}=C_{1}V\\ \frac{k_{3}\psi }{{\left(g+k_{3}\right)}^{2}}\approx \frac{4\gamma_{1}^{2}k_{3}\psi^{3}}{\gamma_{2}^{2}k_{2}^{2}V^{2}}=C_{2}V^{-2} \end{array}$$

the Jacobian at *p*_2_ is given approximately by

(13) $$\text{J}\left(p_{2}\right)\approx \left[\begin{array}{cc} -\gamma_{1} & C_{1}V\\ C_{2}V^{-2} & -\gamma_{2} \end{array}\right]$$

In this case, when *V* is large, the Jacobian has eigenvalues *l*_1_ ≈ −*γ*_1_ and *l*_2_ ≈ −*γ*_2_. Since both eigenvalues are negative, the fixed point *p*_2_ is also stable, with the system decay rate dominated by the decay rates *γ*_1_, *γ*_2_. Finally, the other intersection point *p*_3_ occurs at a concentration $g_{p_{3}}$ below *k*_1_, where the term $V\frac{ng^{n-1}k_{1}^{n}}{\left(g^{n}+k_{1}^{n}\right)}\frac{k_{2}}{k_{2}+h_{p_{3}}}$ may take arbitrarily large (positive) values for large *V*. In this case, at least one of the eigenvalues of the Jacobian must have a positive real part, and the fixed point must therefore be unstable.

We thus conclude that, for small *V*, there is only one (stable) fixed point at the origin; we call this the *monostable regime*. As *V* is increased, two further fixed points emerge, one stable (at high *g*), and one unstable (at low *g*); this is the *bistable regime*. The bifurcation that creates a new pair of fixed points (one stable and the other unstable) is known as a saddle-node bifurcation. Note that changes in other parameters (including *k*_1_,*k*_2_,*ψ*) also result in a similar bifurcation, either by stretching the *g* nullcline or by shifting the *h* nullcline. Therefore, in the following, we will place emphasis on variations in *V* noting that our conclusions on the dynamics will apply equally to variations in other parameters.

#### Dynamical trajectories

Eqs. (5,6) describe an excitable system, where the magnitude of the initial perturbation away from steady-state (*g* = *h* = 0) determines the dynamical trajectories. In the context of the biological system, the stable fixed point at the origin (*p*_1_) represents the case where the gene is unsilenced. From the stability analysis, it follows that small perturbations from this fixed point will decay rapidly and monotonically in *g* (Figure S7a). Larger perturbations result in dynamical trajectories that follow a characteristic long path in phase space, as can be seen in Figure S7b-d. The lower branch of the nullcline, Eq. (8), is a separatrix, so that trajectories that start above it move away from the origin and towards the upper branch of the nullcline, Eq. (8). For these trajectories, it is possible for both *g,h* to increase. The trajectories then progress along the branch, with their fate dependent on whether the system is positioned in the monostable or bistable regime. In the former (stable) regime, the trajectory “drops off the tip” and decays back towards the origin. In the latter (bistable) regime, the trajectory converges on the stable fixed point *p*_2_. Examples of the range of dynamical trajectories are shown in Figure S7.

##### Non-zero production

The analysis thus far focused on the case where there is no production *I*(*t*) = 0 (only autocatalysis). More generally, the production rate may be non-zero in several scenarios. One important case is where production occurs following the presentation of a persistent dsRNA trigger, which underlies much of the experimental work on this system. Production may also be non-zero for endogenous genes, as well as for experimental systems such as multi-copy gene arrays. These systems are associated with the persistent production of dsRNAs (and thus translate to *I*(*t*) > 0)^75^. Persistent (albeit fluctuating) production of dsRNAs may be typical of many genes. Here, we will now study the properties of this more general situation.

When the production *I*(*t*) > 0 is constant *I*(*t*) = *I*, the origin *g* = 0, *h* = 0 is no longer a fixed point of the system. Instead, a stable fixed point appears at *g* ≈ *I*/*γ*_1_, $h\approx \frac{1\psi }{\gamma_{2}\left(I+\gamma_{1}k_{3}\right)}$. The original nullcline at *g* = 0 shifts upwards to *g* ≈ *I*/*γ*_1_, while the second nullcline becomes stretched towards the right. If the system were positioned in the monostable regime, the increase in production rate may potentially lead to a bifurcation with the birth of a pair of stable/unstable fixed points (see Figure S8A,B). In this scenario, the system may alternate between some baseline level of gene silencing and a higher excitable permanently silenced state (similar to the dynamics observed in Klosin et al.^14^). Finally, at even higher rates of production *I*(where *I*/*γ*_1_ > *k*_1_), the shape of the nullcline will change and another bifurcation will occur. In this case, the system will only have a single fixed point at a high silencing level.

Going forward, we will consider the production rate *I*(*t*) as a time-varying (and, occasionally, stochastic) variable. In this case, a step-like increase in *I*(corresponding to treatment with dsRNA trigger) results in a shift of *g,h* towards the upper-right corner of the phase space while, following cessation of dsRNA treatment, the dynamics will either return to *p*_1_ (monostable regime) or shift to *p*_3_ (bistable regime), as depicted in Figure S5.

##### Dynamics near the critical point

As *V* increases, we showed above that the system transitions from a monostable to a bistable regime. This transition occurs at a specific *V* = *V*_crit_ via a saddle-node bifurcation. Near the bifurcation, when *V* is either slightly above or below *V*_crit_, the system behaves in a special manner that will be of crucial importance for this study. Here, we will summarize some aspects of this behavior.

Consider the case where *V* > *V*_crit_ and *V* is slowly decreased until the critical point *V* = *V*_crit_. Remember that the stable fixed point *p*_3_ is associated with positive eigenvalues *l*_1_,*l*_2_ > 0 and that the unstable fixed point *p*_2_ has a negative eigenvalue *l*_1_ < 0. At *V* = *V*_crit_ these points coalesce to a single critical point positioned at *p*_crit_. At *p*_crit_, one of the eigenvalues of Eq. (11) becomes zero (*l*_1_ = 0). This eigenvalue is associated with slow movement parallel to the *g*-axis. The other eigenvalue, *l*_2_ = −*γ*_2_, is associated with rapid convergence parallel to the *h*-axis. The dynamics of the system near the critical point are thus rendered effectively one-dimensional. To analyze the behavior in the vicinity of this point, we can consider the normal form of the saddle-node bifurcation:

(14) $$\dot{x}=x^{2}-\mu$$

where *µ* is the bifurcation parameter corresponding to the scaled distance from bifurcation (Figure S9). Note that, in the case of the TI model, the parameter *µ* essentially captures the distance between *p*_max_ and the nullcline of *h*, which saturates at $h=\frac{\psi }{\gamma_{2}}$. Thus, applied to the current system, *µ* ∝ *V* − *V*_crit_. We will thus consider Eq. (14) to retrieve properties of interest of the system near the critical point, which will greatly simplify the analysis. For *μ* > 0, the normal form of the rate equation has two fixed points: a stable fixed point at $x=-\sqrt{\mu }$ and an unstable fixed point at $x=\sqrt{\mu }$. For *μ* < 0 the system is only stable at *x* = ∞ while, for the critical case *μ* = 0, there is a semi-stable fixed point at the origin. In the original system, *μ* > 0 corresponds to the bistable regime, while *μ* < 0 corresponds to the monostable regime.

A key quantity of interest when the system is in the bistable regime (*μ* > 0) is its response to perturbations near the fixed point $x=-\sqrt{\mu }$. Setting $x'=x+\sqrt{\mu }$, we can linearize the rate equations around the fixed point to obtain:

(15) x˙'=−2μx'

In response to the perturbation, the system decays back to the stable fixed point. However, as *μ* → 0 (that is, as *V* → *V*_crit_), the timescale of the response diverges and perturbations around the fixed point become persistent. This is the phenomenon of critical slowing down - small perturbations around the critical point become long-lived.

Crucially, for *μ* < 0 (the monostable regime), the system may also experience a protracted delay when it transitions near *x* = 0 (a phenomenon known as a “ghost” ^42^). Consider a trajectory starting at some *x*_0_ < 0, which will then move towards *x* → ∞. We can compute the delay by integrating the rate equation around the origin:

(16) $$T=\int_{0}^{T}dt=\int_{x_{0}}^{\infty }\frac{dx}{x^{2}-\mu }=\frac{\tan^{-1}\left(\frac{x}{\sqrt{\left|\mu \right|}}\right)}{\sqrt{\left|\mu \right|}}{|}_{x_{0}}^{\infty }\approx \frac{\pi }{\sqrt{\left|\mu \right|}}$$

Thus, in the monostable regime, as *V* → *V*_crit_, the delay around the fixed point diverges as *T* ∼ |*V* − *V*_crit_|^−1/2^. In the context of the TI model, this means that a large fluctuation from the fixed point *p*_0_ induced by a trigger will follow a trajectory in phase space that incurs a long time delay *T* close to the saddle-node bifurcation before decaying back to *p*_0_.

##### Effect of noise on the dynamics

Noise in molecular reaction dynamics plays an important role in a myriad of biological processes. It may result from intrinsic fluctuations (that may occur, for example, when the number of reacting species is small) or from extrinsic fluctuations due to metabolism, stress, etc. In our case, noise is especially important since, near the critical point, its effects may become amplified. Additionally, in the deterministic (noise-free) model given by Eqs. (5,6), the time delay at *V* = *V*_crit_ is *T* = ∞, while, in a more realistic setting, fluctuations are likely to result in *T* remaining finite. To address the role of fluctuations, we therefore modified Eqs. (5,6) to include noise terms:

(17) $$dg=\left(I\left(t\right)+V\frac{g^{n}}{k_{1}^{n}+g^{n}}\frac{k_{2}}{k_{2}+h}-\gamma_{1}g\right)dt+\sigma_{1}g\;dW_{1}$$

(18) $$dh=\left(\psi \frac{g}{k_{3}+g}-\gamma_{2}h\right)dt+\sigma_{2}h\;dW_{2}$$

where *σ*_1_, *σ*_2_ are the respective noise amplitudes, and *W*_1_, *W*_2_ denote (uncorrelated) one-dimensional Wiener processes ^36^. Here we consider multiplicative noise, which is common in biological settings and has the attractive property that it cannot result in negative concentrations. We note that considering additive noise would lead to identical conclusions. Near the critical point, there is little variation in (*g,h*). Therefore, to study the impact of noise on the dynamics, we may transform Eq. (14) to the corresponding stochastic differential equation:

(19) $$dx=\left(x^{2}-\mu \right)dt+\sigma W$$

where *W* is a one-dimensional Wiener process and *σ* denotes the effective noise amplitude. In the bistable regime, *T* is governed by the escape rate from the stable fixed point at $x=-\sqrt{\mu }$ to the stable fixed point at infinity. In fact, Eq. (19) is much more general - it captures the behavior of a wide class of models undergoing a saddle-node bifurcation in the vicinity of the critical point, including models with nonlinear noise or fluctuations in parameters^43^.

Here, we may make an analogy to the thermally-assisted escape rate of an over-damped particle confined to a well with the potential:

(20) $$\Phi =-\frac{x^{3}}{3}+\mu x$$

The delay time *T* can be estimated by using Kramer’s approximation for the escape rate from a potential well:

(21) $$T\approx \frac{2\pi }{\sqrt{\ddot{\Phi }\left(-\sqrt{\mu }\right)|\ddot{\Phi }\left(\sqrt{\mu }\right)|}}e^{\frac{2\left(\Phi \sqrt{\mu }\right)-\Phi \left(-\sqrt{\mu }\right)}{\sigma^{2}}}=\frac{\pi }{\sqrt{\mu }}\exp\left[\frac{8}{3\sigma^{2}}\mu^{\frac{3}{2}}\right]$$

Thus, as the amplitude increases beyond the bifurcation point *V* > *V*_crit_, there is a steep rise in the escape time *T*, increasing as ln *T* ∼ *μ*^3/2^ (and thus ln*T* ∼ (*V* − *V*_crit_)^3/2^). The approximations for scaling behavior in the bistable regime (*μ* > 0, where ln*T* ∼ *μ*^3/2^ in the noisy case) and monostable regime (*μ* < 0, where *T* ∼ |*μ*|^−1/2^ in the deterministic case) are specific cases for the general case (any *μ*) as shown recently by Hathcock and Sethna^43^. In this case, *T* scales as

(22) $$T\approx 2^{\frac{1}{3}}\pi^{2}\sigma^{-\frac{2}{3}}\left({\text{Ai}}^{2}\left[2^{\frac{2}{3}}\mu \sigma^{-\frac{4}{3}}\right]+{\text{Bi}}^{2}\left[2^{\frac{2}{3}}\mu \sigma^{-\frac{4}{3}}\right]\right)$$

where Ai,Bi denote the first and second Airy functions. The behavior of Eq. (22) resembles the Kramers escape time for the bistable regime and deterministic scaling for the monostable regime, and can be used to study the escape rate for arbitrary *μ* in the vicinity the critical point, regardless of the specific model details. Note that the dimensionless parameter $\xi =\mu \sigma^{-\frac{4}{3}}$ plays an important role in determining silencing duration, as for large *ξ* silencing is effectively infinite.

The incorporation of noise into the dynamical rate equations allows us to consider other statistics of interest, including the variance of sample trajectories around the stable fixed-point in the bistable regime, and the autocorrelation of individual trajectories (defined as the correlation between the values of sample paths over a fixed time-lag, Figure S10). Both quantities diverge near the critical point, and there is extensive literature on the nature of this divergence (as these are considered as “early indicators” for critical transitions ^44^). Here we briefly derive the main results. Around the stable fixed point at $x=-\sqrt{\mu }$ the equation is approximately the Ornstein–Uhlenbeck process:

(23) $$dx\approx -2\sqrt{\mu }\left(x+\sqrt{\mu }\right)dt+\sigma \;dW$$

whose variance is:

(24) $$\mathrm{var}\left(x\right)=\frac{\sigma^{2}}{4\sqrt{\mu }}$$

we can also consider a population of *K* worms, where *x* fluctuats about the fixed point, denoted by time-dependent *K*-dimensional vector *X*(*t*). The autocorrelation is then:

(25) $$\text{cor}\left(X\left(t\right),X\left(t'\right)\right)=e^{-2\sqrt{\mu }\left|t-t'\right|}$$

We can thus see that while there is a divergence of the variance and autocorrelation around the critical point *μ* ≈ 0, as the system transitions to bistability, the variance reduces and fluctuations become short-lived. We note that further away from the critical point, the normal form becomes invalid and one can use the linearization around the fixed point to estimate the dynamics, which shows that the variance and the autocorrelation both become dominated by the large dilution terms. We therefore expect fluctuations to become rapidly suppressed around the stable fixed point, yielding a rapid memoryless ON/OFF-like silencing distribution, rather than a continuum of silencing states.

#### Variation in model parameters around the critical point

We next considered the effect of variation in model parameters on variation in silencing duration when the system is positioned in the vicinity of the critical point. Here, once again, one may take advantage of the normal form of the dynamics of the system near the saddle-node bifurcation, Eqs. (14,19). We can capture the variation by considering *μ* in the normal form of the model as a random variable, drawn from some distribution *F_μ_*(*μ*). To derive analytical results, we can divide the contributions from where *F_μ_* is in the monostable regime (where *μ* < 0), denoted *F*_*μ*−_, and from where *F_μ_* in the bistable regime (where *μ* > 0), denoted *F*_*μ*+_. Starting with the contribution from the former, in this case *T* ≈ *a*|*μ*|^−1/2^ (where *a* is a constant), from which it follows that $\mu =\frac{a^{2}}{T^{2}}$. It therefore follows that the probability density for *T* is given by

(26) $$\mathrm{Pr}\left(T\right)=F_{\mu_{-}}\left(-\frac{a^{2}}{T^{2}}\right)\left|\frac{d\mu }{dT}\right|=F_{\mu_{-}}\left(-\frac{a^{2}}{T^{2}}\right)\frac{2a^{2}}{T^{3}}\approx F_{\mu_{-}}\left(0\right)\frac{2a^{2}}{T^{3}}$$

where the last approximation is relevant for large *T*. Thus, the part of the distribution where *μ* ≪ 0 contributes a delay distribution that scales as *T*^−3^.

When variations in parameter values place the system in the bistable regime, with the distribution *F*_*μ*+_, it follows from the analysis above that $T\approx A\mu^{-1/2}e^{B\mu^{3/2}}$ (with constants *A*, *B*). In this case, the tail of the distribution is dominated by large *μ*. Therefore, to simplify the analytical expressions, we will neglect the *μ*^−1/2^ prefactor and simply take $T\approx Ae^{B\mu^{3/2}}$, or *μ* ≈ (*B*^−1^ln(*T*/*A*))^2/3^. In this case, the probability density is given by

(27) $$\mathrm{Pr}\left(T\right)\approx F_{\mu_{+}}\left({\left(\ln\left(\frac{T}{A}\right)B^{-1}\right)}^{\frac{2}{3}}\right)\left|\frac{d\mu }{dT}\right|\approx \frac{1}{T}\left(F_{\mu_{+}}\left({\left(\ln\left(\frac{T}{A}\right)B^{-1}\right)}^{\frac{2}{3}}\right)\frac{2}{3B}\frac{1}{\sqrt{\frac{\ln\left(\frac{T}{A}\right)}{B}}}\right)$$

The term $\sqrt[{3}]{\frac{\ln\left(T/A\right)}{B}}$ varies slowly at large *T* compared with the 1/*T* dependence. The term $F_{\mu_{+}}\left({\left(\frac{\ln\left(T/A\right)}{B}\right)}^{2/3}\right)$ depends on the form of the distribution. However, typically, it will decay very slowly when the variance of the distribution is large. For example, in the case of a normal distribution, $F_{\mu }=\frac{e^{-{\left(\mu /b\right)}^{2}}/2}{\sqrt{2\pi }b}$, the survival function is then given by

(28) $$S\left(T\right)=\frac{G\left(T\right)}{G\left(T_{\min}\right)}$$

where:

(29) $$G\left(T\right)=\frac{{\left(\frac{\ln\left(T/A\right)}{B}\right)}^{2/3}E_{\frac{1}{2}}\left(\frac{1}{2b^{2}}{\left(\frac{\ln\left(T/A\right)}{B}\right)}^{4/3}\right)}{4\sqrt{2\pi }b}$$

As the standard deviation *b* increases, this function becomes effectively constant for large *T*. (Note that other plausible distributions, such as log-normal, show similar behavior.) In this case, the tail of distribution Pr(*T*) becomes entirely dominated by the 1/*T* dependence, resulting in a heavy tail with a nearly flat survival function (Figure S11).

### Model comparison

#### Developing a simplified minimal TI model for modelling transgenerational silencing in worms

Now that we have reviewed and analyzed the key quantitative properties of the TI model, we next consider how these properties may be reconciled with the observed experimental phenomenology described in Figure 1. To simplify the analysis, we will focus on a simplified version of the TI model based on Eq. (19) (the normal form for a stochastic saddle-node bifurcation). While this equation is strictly valid only in the vicinity of the critical point, it has several advantages for our analysis. First, it captures the relevant trends as the system transitions to monostability or bistability. Second, it is much simpler to analyze, having fewer parameters than the original TI model. Finally, Eq. (19) holds for all systems undergoing a saddle-node bifurcation, so our results can be readily generalized to a much wider class of relevant models.

To apply Eq. (19) as a model of transgenerational inheritance dynamics, we need to define precisely its correspondence with experiments such as the *gfp* silencing experiments reported in Figure 1. In all settings, we consider *x* to define the silencing state of an individual gene, with *x* = *x*_min_ denoting the maximum silencing level and *x* = *x*_max_ as full de-silencing. More generally, we may consider a silencing function *s*(*x*), which increases with *x*. Following a dsRNA trigger, we assume that *x* is initially set at some *x*_0_ = *x*_min_ ≪ 0, while de-silencing occurs at the vicinity of *x*_max_ ≫ 0. To allow for such de-silencing at an absorbing *x*_max_ to occur, in this section we use the modified dynamical equation:

(30) $$dx=\min\left(x^{2}-\mu ,\gamma \left(x_{\max}-x\right)\right)dt+\sigma \left(1-\frac{x}{x_{\max}}\right)dW$$

While, in general, we are interested in transgenerational timescales and ignore specific life-history events (e.g., birth, growth, reproduction, death) in the TI model, reconciling the model with some of the experimental data requires defining these events with respect to the dynamics of the model. This is particularly important when considering de-silencing among sister worms, as we need to set their initial conditions relative to their parent worm. A summary of the model, relative to the life-history of the worms, is provided in Figure S12. We consider the life-history stages of egg-hatching, larval growth, and adult reproduction, with respect to the silencing dynamics of a gene, represented by Eq. (19). Specifically, we consider an individual hatched larva as having an initial state *x* = *x*_0_ and a bifurcation parameter *μ*, with the dynamics of *x* evolving during larval growth according to Eq. (19). Here we assume that the dynamics of the silencing factor as provided by Eq. (19) occur during growth, when dilution is strongest. The adult then transmits its silencing state *x* to all its progeny.

The parameter *μ* (as well as *σ*) could be a function of the gene being silenced, as well as experimental conditions, and there may also be long-term fluctuations within individual worm lineages. As a minimal assumption, we will consider *μ* as drawn from a Gaussian distribution of some width *ρ*, but we will also consider more complicated scenarios where *μ* fluctuates over time to allow for transitions between monostability and bistability. We will assume that sister worms have similar *μ* and *x*_0_. This is motivated by several considerations. Sister worms not only share the environment of their parent, but they also share a syncytium during the crucial larval growth stages. We also expect them to experience similar environmental and experimental conditions. Finally, the observation of Houri-Zeevi et al. of uniform de-silencing amongst sister worms^16^ further motivates similar initial conditions.

Finally, we note that Eq. (19) only captures the dynamics of the silencing trajectories that approach the vicinity of the stable silenced fixed-points, and do not capture the dynamics of silencing trajectories that do not cross the unstable separatrix in Figure S5 - those trajectories simply decay rapidly back to the de-silenced state.

#### Models and criteria for comparison

In the following, we consider models with *μ* < 0 (monostable), *μ* > 0 (bistable), and *μ* ≈ 0 (critical). We will also consider models where there are large fluctuations in parameters values, where worms may transition stochastically between monostability and bistability - we call these mixed models. A mixed model may, for example, correspond to stochastically shutting off the catalysis of silencing factors. In addition, we will consider whether the noise is large (*σ* ≫ 0) or small (*σ* ≈ 0), and whether there is variation in initial conditions. Altogether, this results in 4 ⋅ 2 ⋅ 2 = 16 models.

What is the minimal set of assumptions that is consistent with the known experimental phenomenology? The criteria for model comparison are the following: (a) Can the model provide a silencing duration *T* of several generations (being not very small nor infinite)? (b) Does the model predict a heavy-tailed distribution of silencing duration? (c) Does the model provide a continuum of silencing levels? Can silencing be maintained indefinitely with continuous variation upon selection on this variation? (d) Is the de-silencing of sister worms uniform?

#### Model comparison: silencing duration

The first criteria allows us to rule out all monostable models, as in this case silencing duration would be dominated by dilution and thus be less than one generation (as we expect a dilution of at least two orders of magnitude each generation). This is true regardless of whether noise is large or small, or whether there is variation in initial conditions. For the bistable model, due to the super-exponential dependence of *T* on the dimensionless quantity $\xi =\frac{\mu }{\sigma^{4/3}}$ in Eq. (21), it is required that *ξ* would be set within a narrow range: any increase in the bifurcation parameter *μ* needs to be compensated by an increase in *σ*, otherwise effectively *T* = ∞. We call the first scenario “large *μ* / large *σ*” and the latter scenario “large *μ* / small *σ*”. While the first scenario allows for finite-but-long-term *T*, the second scenario can only create finite *T* if there is variation in the initial conditions, such that some animals do not cross the seperatarix threshold for bistability. In this case, consider the distribution of silencing duration Pr(*T*) when there is variation in initial conditions. From the properties of the TI model, when *g*_*t*=0_ is below some critical value, silencing dynamics falls in the monostable regime and *T* < 1, while when *g* is above this critical value, the system transitions to silenced fixed point and silencing duration is effectively infinite *T* ≈ ∞. This evident from Figure S13, which depicts one-generation trajectories in the g-h space starting from different concentrations of g - all trajectories effectively de-silence or transition to the stable fixed point in a single generation.

For the critical regime (*μ* ≈ 0) we need to have a small *σ* to allow for long-term silencing (again due to the dimensionless quantity *ξ*). We call this the “small *μ* / small *σ*” model. Finally, for the mixed model, it is possible to generate a long-term *T* if the transitions from bistability to monostability occur rarely. This may correspond, for example, to worms transiently shutting off the catalysis of silencing factors once every few generations on average.

The first criteria of finite and long-term silencing duration thus results in the following models: bistability with large noise, critical regime with low noise, and a mixed model with rare transitions from bistability to monostability.

#### Model comparison: distribution of silencing duration

Recall that for a fixed *μ*, *σ*, silencing duration is given by Eq. (22), which grows as a super-exponential for *μ* > 0 (viz. $e^{b\xi^{1.5}}$ with *ξ* ∝ *μ*) but decays like a power law for *μ* < 0 (viz. |*ξ*|^−1/2^). Furthermore, recall that for both the large *μ* / large *σ* model, and the small *μ* / small *σ* model, *ξ* is small, but for the former *μ* ≫ 0 while for the latter *μ* ≈ 0. Finally, recall that *μ* itself relates to the distance of parameters from the bifurcation, e.g., *μ* ∝ *V* − *V*_crit_.

For all models, when parameters are fixed precisely, we do not expect to see heavy-tailed distributions. For the mixed model (with memoryless transitions), as well as for models with noise driven escape from the stable silenced state, we expect that the distribution of silencing duration would be exponential, corresponding to memoryless de-silencing. When *μ* < 0, we expect to see a narrow (Gaussian-like) distribution of silencing duration ^43^. To explain the heavy-tailed variation in silencing duration, we must therefore consider the possibility of variation in parameters between genes and worm lineages.

For the mixed model, variation in parameters is also not expected to generate heavy tails, as the tail would become dominated by the slower transition rates (Figure S14). Variation in parameters is also less likely to be consequential for the high *μ* / high *σ* model (Figure S14). To see why this is the case, consider that, in this case, silencing duration is roughly proportional to $e^{b\xi^{1.5}}$, with $\xi =\frac{\mu }{\sigma^{4/3}}\propto \frac{V-V_{\text{crit}}}{\sigma^{4/3}}\approx \frac{V}{\sigma^{4/3}}$ (where the latter is due to both *V*, *σ* being large in this model). Thus, small relative variation in *V* translates only to proportionally small variation in *ξ*, limiting the consequence of the variation on silencing duration.

On the other hand, for the model with low *μ* / low *σ*, we have *V* ≈ *V*_crit_ and *σ* ≈ 0. Small variation in *V* can therefore become greatly amplified, with genes with slightly higher *V* having much longer silencing duration. This is best illustrated when considering the zero-noise case *σ* = 0 - in this case, *T* > 1 generation at some *V* ≈ *V*_crit_, but *T* = ∞ at *V* ≥ *V*_crit_. In fact, variation in *μ* around the critical point captures precisely the shape of the heavy-tailed distribution presented in Figure 1. (Figure S14)

#### Model comparison: selection and uniform de-silencing among sister worms

Both the large *μ* / large *σ*, and the small *μ* / small *σ* models have large, continuous variation around the silenced state (see Eq. (24)). However, this variation is of very different nature in the two scenarios, due to the different autocorrelation of the variance (see Eq. (25)). For the large *μ* case, fluctuations are short-lived, with trajectories reverting rapidly back to the stable fixed point. Large fluctuations can cause the silencing state to cross the unstable fixed point, and this would be followed by rapid de-silencing. De-silencing is therefore effectively a memoryless process on the transgenerational timescale. When *μ* is small, on the other hand, the large variance is due to critical slowing down, where fluctuations become long-lived. Small fluctuations in silencing factors, or parameters, as well as small variations in initial conditions, lead to persistent variation in the silencing state at the population level. These differences are illustrated in Figure S15, where sample trajectories are plotted for the dynamics in the monostable, critical, and bistable regimes.

These differences are consequential for understanding the effects of selection, as well as de-silencing among sister worms. For selection, consider selecting worms with stronger silencing at a certain time point, and then observing the silencing levels of their offspring in the following generation. When *μ* is large, the variation corresponds to short-lived fluctuations, which we expect to be rapidly eliminated, and have little consequence in the next generation due to the memoryless nature of the de-silencing process.

On the other hand, when *μ* is small, selection can be highly effective. Stronger silencing corresponds to individuals where the silencing state is more negative (*x* < 0), while weaker silencing would correspond to individuals with a silencing state *x* ≈ 0 or *x* > 0. Moreover, given that there is some fluctuating variation in *μ*, individuals with weaker silencing may be closer to the monostable regime, while those with stronger silencing stay closer to the bistable regime. Due to the slow decay of the autocorrelation of silencing levels and static progression around *x* = 0, we expect that individuals with stronger silencing would progress only slightly in *x* over a single generation, avoiding de-silencing. Thus, selecting those individuals may allow silencing to be maintained indefinitely without de-silencing events.

Notice that monostability (even with slow dynamics) is not consistent with effective selection, as the variation is not static, and we expect the average *x* to change over time.

Similar considerations suggest that only the small *μ* mechanism can explain the uniform de-silencing among sister worms. To see why, consider an individual worm that has been de-silenced. In the large *μ* case, due to the memoryless nature of the de-silencing process, de-silencing can occur rapidly from the stable fixed point. It is therefore likely that, among sister worms that start their life with *x* around the stable fixed point, some will become de-silenced, while others will not. In the small *μ* case, on the other hand, due to slowdown, de-silencing is preceded by a long transient in a state *x* to the right of the critical point. The dynamics from this starting point are like the monostable case, where there is monotonic progression in phase-space, so we expect to see uniform de-silencing among sister worms.

These effects can be illustrated by considering the dynamics of *x* in the critical regime when the initial conditions are varied (Figure S16). During the long delay around the critical region, it is possible to “stop” the distribution by selecting worms with stronger silencing (lower *x*). However, if higher *x* is selected, the dynamics will monotonically decay towards (uniform) de-silencing.

#### Model comparison: summary

Taken together, comparison with the known experimental phenomenology supports a model where the system is positioned close to a saddle-node bifurcation (*μ* ≈ 0) with relatively little noise (small *σ*). We therefore propose that, for the transgenerational inheritance of gene silencing in worms, the system is positioned near *μ* = 0. Small noise (*σ*) and small fluctuations and variations in *μ* produce a range of silencing durations, from fast de-silencing (1 generation) to long-term silencing, as well as intermediate silencing durations, with a typical heavy-tailed distribution. After the dsRNA trigger is placed, the system progresses rapidly towards the vicinity of the bifurcation point (*x* = 0) (see Figure S16). Selection of worms with stronger silencing (smaller *x*) maintains a static steady-state distribution. However, upon random selection, some selected worms have slightly weaker silencing (larger *x*). Those worms would transition from the critical region to the region where there is rapid monostable decay towards de-silencing. This transition is uniform in its final stages, providing uniform de-silencing of sister worms.

### Toggle-Inhibitor-Competition (TIC) model

Close to the saddle-node bifurcation, the TI model provides a framework to describe much of the observed phenomenology of gene silencing. However, as discussed in the main text, its application to the experimental system requires a high degree of fine-tuning, which is hard to motivate. Here, we develop a modified framework based on competition for silencing machinery, in which the system self-organizes around the saddle-node bifurcation without fine-tuning.

In the Toggle-Inhibitor model, emphasis was placed on the silencing of a single gene. The Toggle-Inhibitor-Competition (TIC) model considers the dynamics of all genes, accounting for their competition over shared synthesis resources. Let *i* = 1, …, *N* denote the indices of genes in the ensemble of endogenous and exogenous genes that may become silenced, with the corresponding concentrations of gene-specific siRNAs and silencing modification marks denoted by *g_i_*, *h_i_*. The collective dynamics are then given by the coupled set of rate equations

(31) $$\dot{g}=I_{i}+\frac{V_{\text{tot}}}{C\left(\left\{g_{i},h_{i}\right\}\right)}\frac{g_{i}^{n}}{k_{1}^{n}+g_{i}^{n}}\frac{k_{2}}{k_{2}+h_{i}}-\gamma_{1}g_{i}$$

(32) $${\dot{h}}_{l}=\psi \frac{g_{i}}{k_{3}+g_{i}}-\gamma_{2}h_{i}$$

where the “cost” function *C* is proportional to the overall abundance of siRNAs,

(33) $$C=\sum_{i=1}^{N}c\left(g_{i},h_{i}\right)=\sum_{i=1}^{N}g_{i}$$

Here, for simplicity, we have taken the model parameters to be gene-independent, turning later to consider the effect of variations. As we will see below, the precise form of *C* is not important for the conclusions. It is only important that *C* increases with the number of actively silenced genes. Moreover, alternative models where other parameters are modulated, such as a model where *k*_1_ increases with *C*, behave in an almost identical manner for the considered properties of interest.

In this model, we consider the ensemble of production rates *I_i_* as a random variable, where stochasticity may arise due to fluctuating environmental conditions, or internal stochastic events such as transcriptional bursting. To simplify the analysis, we will consider a random process where, at a given generation, *I_i_* = 0 with probability 1 − *q* or *I_i_* = *L* (for a fixed *L* > 0) with probability *q*. Further, with silencing events considered as infrequent, we may assume that *q* ≪ 1. We also assume that silencing events occur only when the gene is unsilenced; that is, when *g_i_*, *h_i_* are near the origin. We note that, for large *N*, fluctuations in *C* due to such stochastic dynamics will be small. Finally, like Eqs. (17,18), in the presence of noise, the corresponding equations take the form:

(34) $$dg_{i}=\left(I_{i}+\frac{V_{tot}}{C\left(\left\{g_{i},h_{i}\right\}\right)}\frac{g_{i}^{n}}{k_{1}^{n}+g_{i}^{n}}\frac{k_{2}}{k_{2}+h_{i}}-\gamma_{1}g_{i}\right)dt+\sigma_{1}g_{i}\;dW_{i_{1}}$$

(35) $$dh_{i}=\left(\psi \frac{g_{i}}{k_{3}+g_{i}}-\gamma_{2}h_{i}\right)dt+\sigma_{2}h_{i}\;dW_{i_{2}}$$

#### Global dynamics and steady state

To analyze the stability of Eqs. (31, 32), we will consider a system starting from either (i) very few silenced genes (and therefore a very small cost *C* = *C*_min_), or (ii) many silenced genes (with very large *C* = *C*_max_). As before, we denote by *V*_crit_ the (this time per-gene) synthesis rate at which the system transitions from monostability to bistability. In case (i), the effective synthesis rate $\frac{V_{\text{tot}}}{c_{min}}$ may be very large, placing the system in the regime of bistability $\left(\frac{V_{\text{tot}}}{c_{\text{min}}}>V_{\text{crit}}\right)$. Thus, new stochastic silencing events (*I_i_* = *L*) will result in stable silencing, driving an increase in the cost function $\left(\dot{C}>0\right)$. On the other hand, in case (ii), the system is in the monostable regime $\left(\frac{V_{\text{tot}}}{c_{\text{max}}}>V_{\text{crit}}\right)$, where genes gradually become silenced and the cost function will fall $\left(\dot{C}<0\right)$. Thus, over time, the system will converge towards a dynamic steady-state (with $\dot{C}=0$) at some intermediate value of the cost function *C* = *C*_st_.

What is the steady-state value *C*_st_? We will now show that, for a wide range of parameter values, *C*_st_ ≈ *C*_crit_, where

(36) $$\frac{V_{\text{tot}}}{C_{\text{crit}}}=V_{\text{crit}}$$

To see why this is the case, suppose that the (average) number of silenced genes at steady state is given by *M* < *N*. Let us denote by *λ* the arrival rate of new silencing events, where according to the model, *λ* = *q*(*N* − *M*). At steady state the system must adhere to Little’s law^54^:

(37) M=λT=q(N−M)T

where *T* is the average silencing duration of an individual gene. In other words, the flux of new silenced genes must equate to the number that become de-silenced. When the system is near the critical point,

(38) $$C_{\text{crit}}\approx Mc_{\text{crit}}$$

where *c_crit_* = *c*(*p*_crit_) is the cost for an individual gene at the stable fixed point (here *c*(*p*_crit_) = *g*_crit_). Combining Eqs. (36-38), we obtain an (approximate) equation for T as

(39) $$T=\frac{1}{q\left(\frac{Nc_{\text{crit}}V_{\text{crit}}}{V_{\text{tot}}}-1\right)}$$

To gain some intuition for Eq. (39), consider the case where *N* = 1000 genes and *q* = 0.01 per generation, so that the arrival rate of new silencing events, when no genes are currently silenced, is 10 genes per generation. Setting *c*_crit_ = 1, *V*_crit_ = 1 (note that these depend on circuit parameters), we obtain the equation, $T=\frac{100V_{\text{tot}}}{1000-V_{\text{tot}}}$. For *V*_tot_ > 10 and *V*_tot_ ≪ 1000, the right-hand side increases linearly with *V*_tot_ and is on the order of several (to several dozens of) generations. This equation will be satisfied near the critical point. The reason for this is that this timescale is slower than the typical timescale in the monostable regime, yet it will be realized in the vicinity of the critical point due to the rapid increase in *T* in this region (see Eq. (21)). More generally, taking the limit *N* → ∞ and *q* → 0 (with *λ* = *qN* constant),

(40) $$T=\frac{V_{\text{tot}}}{\lambda c_{\text{crit}}V_{\text{crit}}}$$

In this limit, any *V*_tot_ > *λc*_crit_*V*_crit_ will result in a transgenerational timescale, which would lead to the system converging to a steady state positioned near the critical point. Finally, from Eqs. (36,38), it follows that

(41) $$M=\frac{V_{\text{tot}}}{c_{\text{crit}}V_{\text{crit}}}$$

so that the average number of silenced genes at steady-state increases linearly with *V*_tot_.

It is important to note that there were certain assumptions made in the above derivations. We assumed a large separation of timescales between that of the steady-state, Eq. (39)), and the rapid turnover timescale in Eqs. (5,6) and the generalization Eqs. (34,35). We also assumed that the system is in the monostable regime when *C* = *C*_max_ and bistable when *C* = *C*_min_. This might not be the case if, for example, there is a large decrease in *N* (as may occur, for example, when piRNAs are deleted). In this case, the system may settle in the bistable state, as can be seen in Figure S17.

##### Variation in parameters

Finally, we turn to consider the role of potential variation in the model parameters. Consider a system that is organized around some steady-state cost *C* = *C*_st_. So far, we assumed that all genes have identical parameters, and that *C* is constant at steady state. However, in a realistic setting, this assumption may break down. One possibility is that there is variation in biochemical parameters between genes, which may be static over time. Another possibility is that there are global fluctuations, such as fluctuations in *V*_tot_, which can lead to variation in *C*_st_ over time and possibly between worms and worm lineages. Another source of variation can be changes in *C*_st_ due to variation in the arrival of silencing memories. This variation can affect both the steady-state cost *C*_st_, as well as the silencing duration of individual genes.

For global and stochastic fluctuations in parameters values, we would still expect that the system will settle near the critical point due to the same argumentation as in previous sections, even in the presence of variation in parameters and between genes. Settling near the bifurcation is a robust property that holds for a wide range of parameter values, and so will be unaffected by fluctuations in global parameter values. For variation in individual genes, we should consider that Little’s law must hold for the genes that are transgenerationally silenced (as they contribute to the cost *C*). As an illustrative example, we can consider a population of genes where half of the genes have a much smaller value of *V*_tot_ than the other genes. In this case, at steady state, the genes with the higher *V*_tot_ will satisfy Eq. (40) (with an arrival rate of *λ*/2), while the genes with the lower *V*_tot_ will be in the monostable regime and thus will contribute only little to *C*. The system will thus settle near the critical point for the genes that have stable transgenerational inheritance.

To extend this argument, we can ask how continuous variation in model parameters affects the global steady-state of the system (Figure S18). To this end, we considered the situation in which *V*_tot_ is Gaussian distributed in the population of genes, with an increasing standard deviation *b*. As can be seen in Figure S18, as *b* increases, the value of $\frac{V_{\text{tot}}}{C}$ decreases slightly below the critical value, and the value of $\frac{V_{\text{tot}}}{C}$ of the silenced genes remains near the critical *V*_crit_. Thus, variation between genes leads to a subgroup of genes closer to the monostable regime (which cannot be transgenerationally silenced), and another subgroup that has longer transgenerational silencing. Similar results hold for variation in all model parameters. Tuning near the critical point is thus robust to variation in parameters between genes.

An intriguing possibility is that the stable variation between genes can be utilized by the silencing system for differential silencing of genes according to a-priori heuristics (akin to innate immunity). Genes which are more likely to correspond to dangerous elements such as viruses may have differentiating motifs (compared with endogenous genes) which would translate into parameters closer to the bistable regime, and thus to long-term silencing. This is made possible by the proximity of the system to the critical regime.

#### More complex model that incorporates pUGylation dynamics

While our analysis focused on the simple two-dimensional TI model (provided by Eqs. (5,6)), our conclusions regarding self-tuning near the critical point apply to a much wider class of models. The only necessary ingredient is the competition over autocatalysis resources between the (excitable) silencing memories, which tune a control parameter for a saddle-node bifurcation. The dynamics can be given by a complex and high-dimensional interaction, rather than by the simple two-dimensional system provided by the TI model. To demonstrate this, we show that our conclusions are effectively unchanged when we consider a more complex model for silencing dynamics, which incorporates the important process of pUGylation of template mrNAs. It has been demonstrated that siRNAs are produced from template mRNAs which have been tagged with poly(UG) tails^65^. The tagging itself is performed in a manner which is directed by existing siRNAs ^65^; it is thus an autocatalytic process. Additionally, we assume that the production of template mRNAs is inhibited by the silencing chromatin marks. Based on these interactions, we propose the following rate equations:

(42) $$dg_{i}=\left(I_{i}+\frac{V_{tot}}{\sum g_{i}}y_{i}-\gamma_{1}g_{i}\right)dt+\sigma_{1}g_{i}\;dW_{i_{1}}$$

(43) $$dh_{i}=\left(\frac{g_{i}}{g_{i}+k_{3}}-\gamma_{2}h_{i}\right)dt+\sigma_{2}h_{i}\;dW_{i_{2}}$$

(44) $$du_{i}=\left(\frac{k_{2}}{h_{i}+k_{2}}-\gamma_{3}u_{i}\right)dt+\sigma_{3}u_{i}\;dW_{i_{3}}$$

(45) $$dy_{i}=\left(\frac{{\left(g_{i}u_{i}\right)}^{n}}{k_{1}^{n}+{\left(g_{i}u_{i}\right)}^{n}}-\gamma_{4}y_{i}\right)dt+\sigma_{4}y_{i}\;dW_{i_{4}}$$

where *g_i_*, *h_i_*, *u_i_*, *y_i_* correspond to siRNA concentration, concentration of chromatin silencing marks, mRNA levels, and mRNA molecules tagged by pUG tails for gene *i* (*i* ∈ 1, …, *N*). As before, we simulated the model with stochastic activation effects. Despite the added complexity, this model also self-tunes near the saddle-node bifurcation point (Figure S19).

#### Generality of global feedback to autocatalysis

In the TIC model, we considered feedback in the form of a cost function *C*({*g_i_*, *h_i_*}) that inhibits autocatalysis by, e.g., normalizing the maximum autocatalysis rate $V=\frac{V_{\text{tot}}}{c\left(\left\{g_{i},h_{i}\right\}\right)}$. One can translate this feedback into a dynamical equation for *V*:

(46) $$\dot{V}=\gamma_{V}\left(V_{\text{tot}}-C\left(\left\{g_{i},h_{i}\right\}\right)V\right)$$

Since *C*({*g_i_*, *h_i_*}) increases with *M*, one can consider Eq. (46) (together with the TI model equations) as a form of proportional feedback from *M* to *g*. As we have shown, together with the stochastic silencing, this results in self-tuning around the critical point with a typical delay that increases in proportional to *V*_tot_. Similar dynamics can be written down for negative feedback through the half-way saturation point *k*_1_.

While proportional feedback is common in biological settings, other forms of feedback are also possible. Specifically, integral feedback is common in biological systems^76,77^, from bacterial navigation^78,79^ to endocrine circuits^80–82^. Such a feedback mechanism can be implemented through a dynamical equation of the form:

(47) $$\dot{V}=\gamma_{V}\left(V_{\text{tot}}-C\left(\left\{g_{i},h_{i}\right\}\right)\right)$$

Eq. (47) is called integral feedback since it integrates over an “‘error’’ in *C* (or effectively over an “error” in *M*). It also results in self-tuning around the critical point. To see why this is the case, consider the steady state of Eq. (47). Steady state is reached only when *V*_tot_ = *C*({*g_i_*, *h_i_*}), which around the critical point provides the constraint:

(48) $$V_{\text{tot}}=c_{crit}M$$

Together with Little’s law (Eq. (37)), and with the steep dependence of *T* on *V* around the critical point, we expect the system to stabilize around the critical point from the same considerations provided for the proportional feedback case, with a linear dependence of *T* on *V*_tot_.

More generally, we can consider any feedback (on *V* or *k*_1_) which results in an algebraic constraint of the form *M* = *z*(*g*, *h*), where *z* is some function. Evaluating *z* at the critical point provides the constraint *M* = *z*(*g*_crit_, *h*_crit_) = *z*_crit_. For a large enough *z*_crit_, we would expect a stabilization around the critical point, since *T* can acquire large values only within a narrow range of *V*, *k*_1_ around the critical point.

#### Fluctuations in circuit parameters

So far, we have considered fluctuations in the abundances of the levels of *g*, *h*. More generally, we can consider fluctuations in model parameters such as *V*, *k*_1_, *k*_2_, etc. All of the results derived so far hold in this more general case, as they depend only on the scaling behavior around the critical point, which holds for arbitrarily nonlinear noise functions ^43^. To illustrate this important point, consider a revised TI model, Eqs. (17, 18) where the dynamics of *V* are defined by the Ornstein–Uhlenbeck process:

(49) $$dV=\gamma_{V}\left(V_{0}-V\right)dt+\sigma_{V}\;dW_{3}$$

where *W*_3_ is a Wiener process, *V*_0_ is the mean value of *V*, and *σ_V_* is the noise magnitude. Repeating the simulations from Figure 3 with the additional equation, we find that, even with the addition of the fluctuations in *V*, there is still a steep super-exponential dependence of silencing duration *T* on the parameter values (in this case *V*_0_) (Figure S20).

This steep dependence is at the heart of the arguments advanced in this study, including the case of tuning near the critical point, as well as the proposed mechanism based on competition between silencing memories. Therefore, it follows, for example, that the TIC model, Eqs. (31, 32), with the additional equation (Eq. (49)) also becomes self-tuned around the critical point.

#### Memory system timescales

The model has two important timescales. The first timescale is the fast timescale of dilution/turnover of memory components, given by the decay times $\gamma_{1}^{-1}$, $\gamma_{2}^{-1}$. The second timescale is the slow memory duration timescale *T*. These timescales govern dynamical properties of the memory system. Consider a step change in a (global) model parameter, such as the overall synthesis capacity *V*_tot_, which will result in a change in the steady-state memory pool size *M*. If this is a down-step (and *M* decreases), the system will be transiently in the monostable regime, and genes will be removed according to the fast dilution timescale (Figure S21). On the other hand, an up-step in *V*_tot_ will result in the system transiently moving to the bistable regime. Genes will then re-accumulate according to the stochastic arrival rate *λ*, with a timescale given by $\frac{M}{\lambda }=T$. Measuring these timescales may be possible in experiments where silencing is known to be globally perturbed, such as in conditions of stress.

#### Arrival-rate is independent of memory duration

A final intriguing possibility is that the dependence on memory arrival rate *λ* in Eq. (40) can be completely removed by adding a feed-forward interaction between the arrival of a new memory event and synthesis capacity *V*_tot_, such that *V*_tot_ = *ηλ* for some proportionality constant *η*. This may occur, for example, if external triggers or internal activation pulses also encourage the production of more autocatalysis enzymes. In this case, Eq. (40) becomes:

(50) $$T=\frac{\eta }{\lambda c_{\text{crit}}V_{\text{crit}}}$$

which depends only on internal biochemical parameters.

### Details of numerical simulations

To simulate model equations, we used either the Euler method (for deterministic ODEs) or the Euler-Maruyama method (for SDEs), taking *dt* = 0.1hr. All other parameters are provided in Tables 1-3. Silencing strength was estimated by taking $s=\sqrt{g^{2}+h^{2}}$, and a gene was considered silenced if *s* > *s*_silenced_. For selection experiments, a gene would be strongly silenced if *s* > *s*_select_. Parameter values are provided in Table S3.

#### Simulations of directed vs. random selection

To simulate experimental selection (as in Figure 3) we considered the setting where, in each generation, *N*= 250 individual worms are simulated. To simulate the transfer of random offspring to new plates, at the end of the generation, another *N*= 250 sister worms were sampled by selecting mother worms with replacements, either from the entire population (random selection) or from worms with strong silencing (directed selection). For simulating sister worms, only a single mother worm was sampled.

#### Simulations of TIC model

The TIC model with stochastic arrivals was simulated by taking a fixed mean arrival rate of new silencing events, given by *λ*, resulting in a Poisson distribution of silencing events per generation. Silencing events were simulated by adding silenced genes with *g* = 3, *h*= 0. Silenced genes were removed from the simulation once they became de-silenced.

#### Simulation parameters

Simulation parameters (including amplification rates and noise magnitudes) are specified in Tables 1-3. Note that the important phenomena associated with the model - namely self-tuning near the critical point - is a generic property that holds for a wide range of parameters and noise levels.

## Supplementary Material

movie s1

movie s2

movie s3

movie s4

movie s5

movie s6

movie s7

Supplementary Material

## Data Availability

All data generated are available from the authors upon request. The code for the simulation of the TI and TIC models, including selection and population dynamics, is available at https://zenodo.org/badge/latestdoi/554841318. No additional information.
