# Supplementary material for: Epigenetic inheritance of gene-silencing is maintained by a self-tuning mechanism based on resource competition
Source: Cell Syst. Author manuscript; Available in PMC 2023 Aug 4. (PMC7614883; doi:10.1016/j.cels.2022.12.003)
Supplement: Supplementary Material [file EMS182032-supplement-Supplementary_Material.pdf]

Supplemental Video Legends

Movie S1: **Tuning of silencing duration near critical point. Related to Figure 2.** Simulations of model Eqs. 1-2 (black lines, parameters given in Table S1) for different values of V (left to right: V=0.45, V=0.55, V=0.8), plotted against model nullclines as in Figure 2. In the monostable regime (left) silencing diminishes rapidly, or diverges to infinity in the bistable regime (right). Near the critical point (middle), however, it is possible to tune silencing time over longer periods.

Movie S2: **Noise allows for escape from mild bistabiltiy. Related to Figure 2.** Simulations of stochastic model equations (black lines, parameters given in Table S1, taking $\sigma_1 = \sigma_2 = 0.03\text{hr}^{-0.5}$) for different values of *V* (left to right: V=0.45, V=0.57, V=0.8), plotted against model nullclines as in Figure 2. The behavior in strong monostability (left) and strong bistability (right) is like the deterministic case, however noise allows for escape from mild bistability (middle).

Movie S3: **Critical slowing-down, related to Figures 2-3.** Simulations of 20 sample paths from stochastic model equations for different values of *V* (left to right: V=0.45, V=0.55, V=0.8), plotted against model nullclines as in Figure 2. A given timepoint of the simulation is plotted as a moving dot, with a 'tail' representing the previous 6 hours.

Movie S4: **Large, static variation allows for efficient selection in critical regime. Related to Figure 3.** Simulations of random selection (left panel) vs. directed selection (right panel) plotted in phase-space.

Movie S5: **In the TI model, the amplification parameters V, k_1 are control parameters for saddle-node bifurcation. Related to Figure 4.** Model nullclines and fixed points (solid for stable fixed points and empty for unstable fixed points) plotted for variation in V (left) or k_1 (right).

Movie S6: **Self-organization near saddle-node bifurcation in the TIC model. Related to Figure 4.** Simulation of the TIC model, starting with N=0 silenced genes. The size of the silenced gene pool *M* increases until it stabilizes (left). The stabilization of the silenced gene pool size *M* is accompanied by organization near a critical point of the model parameters for the silencing of an individual gene (left), as can be seen from the plotting of the model nullclines.

Movie S7: **Variation in circuit parameters leads to variation around the critical point in the TIC model. Related to Fig. 4.** Simulation of the TIC model, taking a 10% variation in V_{tot} between silenced genes. The nullclines of each of the silenced genes is plotted, demonstrating the variation that leads to heavy-tailed silencing duration distributions.

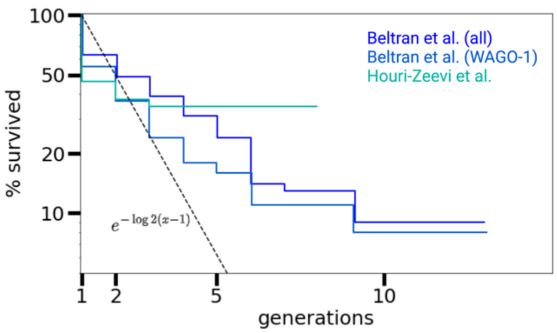

Figure S1: **Log-linear survival curves for silencing events. Related to Figure 1.** Distribution of silencing times from Figure 1E, plotted on a log-linear scale to emphasize the heavy-tailed nature of the experimental data.

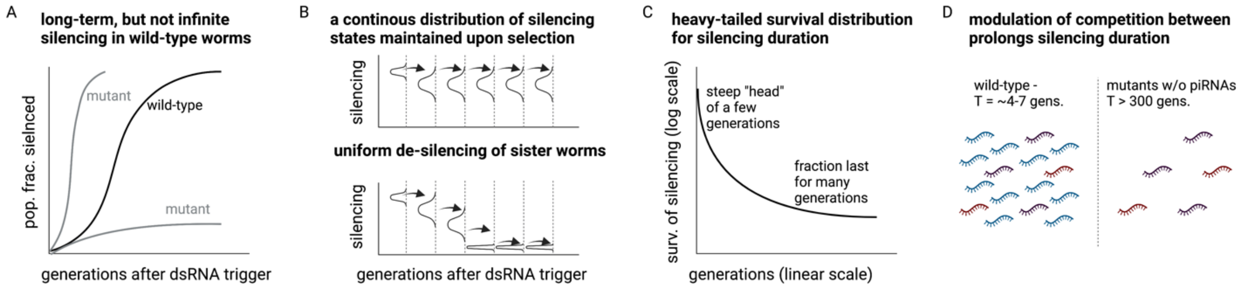

Figure S2: **Schematic illustrations of the experimental phenomena depicted in Figure 1. Related to Figure 1.** (A) Silencing of a gene persists in the population for several generations. Some mutants can show effectively infinite silencing, while others de-silence within a single generation. (B) Following the administration of a silencing trigger, worms become silenced and lose silencing over the timescale of several generations. Among silenced worms there is variation in silencing levels. Selecting the offspring of worms with stronger silencing allows silencing to be maintained for many (>80 generations), with a continuum of silencing levels re-established in every generation. Random selection, on the other hand, results in de-silencing, with de-silencing occurring uniformly amongst sister worms. (C) The distribution of silencing durations of different worn lineages, as well as different genes, shows a typical heavy-tailed distribution. Many genes are de-silenced with a few (1-3) generations, while a small subset can maintain silencing for many (over 7) generations. (D) The potency and duration of the silencing response is associated with competition between silenced memories. This is most evident in mutants where there is a large down-regulation of endogenous small RNAs, including mutants that lack piRNAs - these mutants show stable silencing.

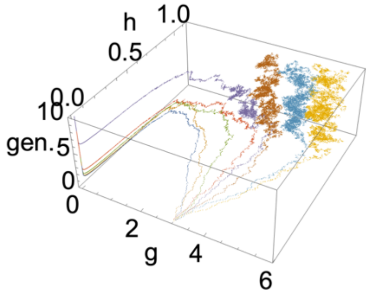

Figure S3: **Dynamics of sample trajectories in the g-h-time space. Related to Figure 2.** Sample trajectories for simulations of the TI model, taking monostable $V = 0.2, 0.3, 0.4, 0.5$ (blue, orange, green, red), critical $V = 0.55$ (purple), and bistable $V = 0.6, 0.7, 0.8$ (brown, cyan, yellow). Trajectories are plotted in the g-h-time space. In the monostable regime there is rapid traversal of the phase-space, while in the bistable regime there are rapid fluctuations around the stable silenced fixed-point. In the critical regime, on the other hand, there is a long delay with slow fluctuations around the stable silenced fixed-point.

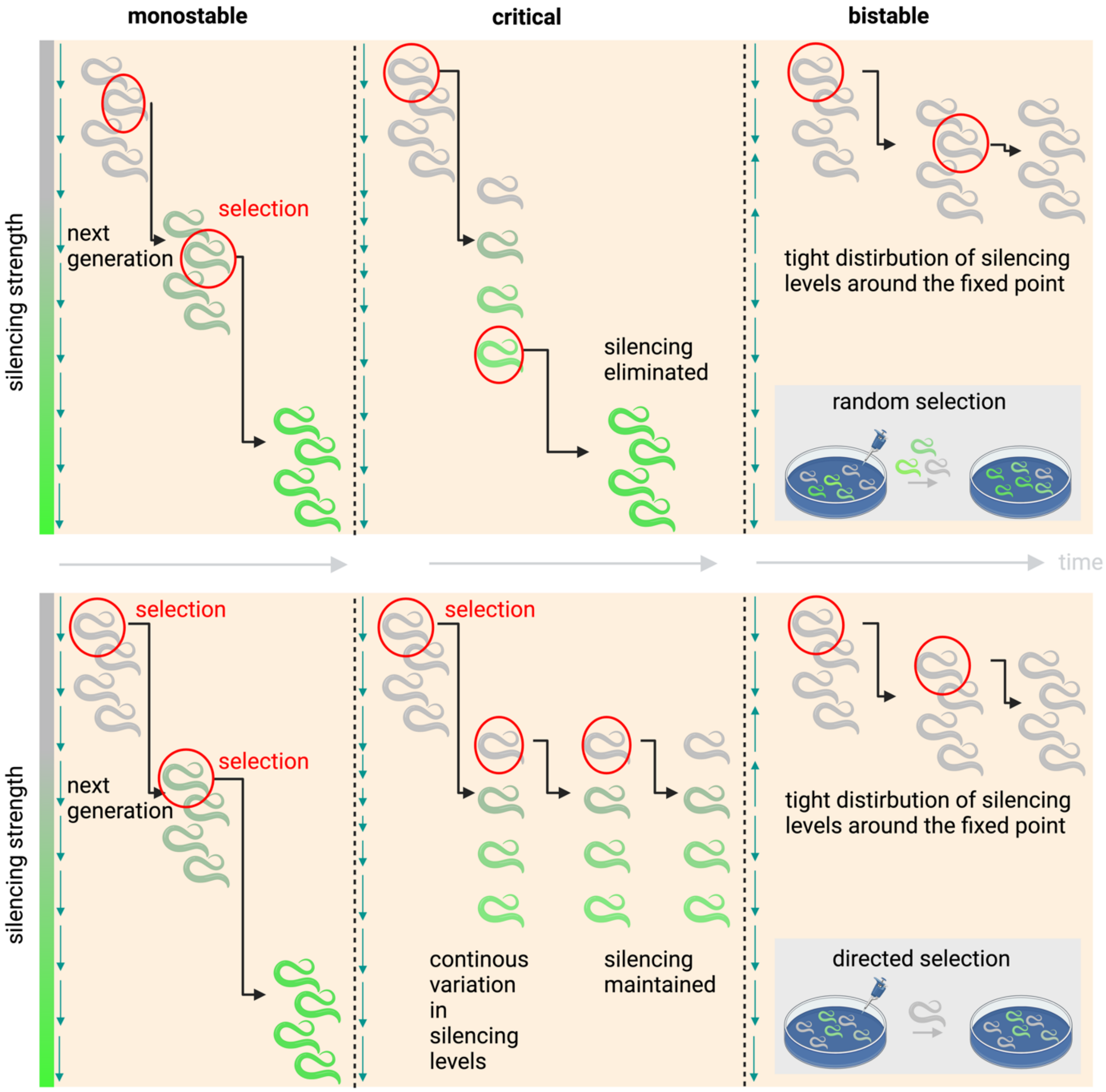

Figure S4: **Schematic illustration of the differential effects of random (upper panel) vs. directed (lower panel) selection. Related to Figure 3.** Here, we consider the effects of selecting worms according to silencing levels in the three major regimes of the system: monostability, the critical regime and bistability. In the critical regime, due to large static variation around the bifurcation point, there is high differential sensitivity to selection.

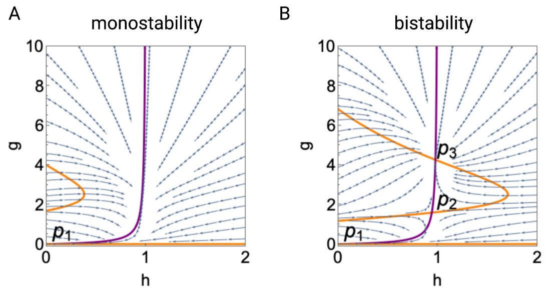

Figure S5: **Stream plots of Eqs. (5,6) and nullclines for $\dot{g} = 0$ (orange) and $\dot{h} = 0$ (purple). Related to Figure 2.** Depicted with parameters as in Table 1, and (A) $V = 0.45$ AU/hr or (B) $V = 0.7$ AU/hr. For (A), the nullclines intersect at $p_1 = (0,0)$ placing the system in a monostable regime. For (B), the nullclines intersect at three points, p_1, p_2 and p_3, placing the system in the regime of bistability.

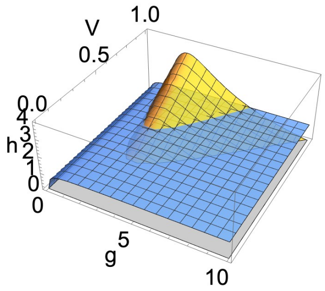

Figure S6: **Three dimensional depiction of the saddle-node bifurcation of the TI model. Related to Figure 2.** Plot of Eqs. (8,9) as surfaces, with the blue surface corresponding to the h-nullclines and the orange surface corresponding to the g-nullcline. Each surface depicts the values of h for different values of g, V.

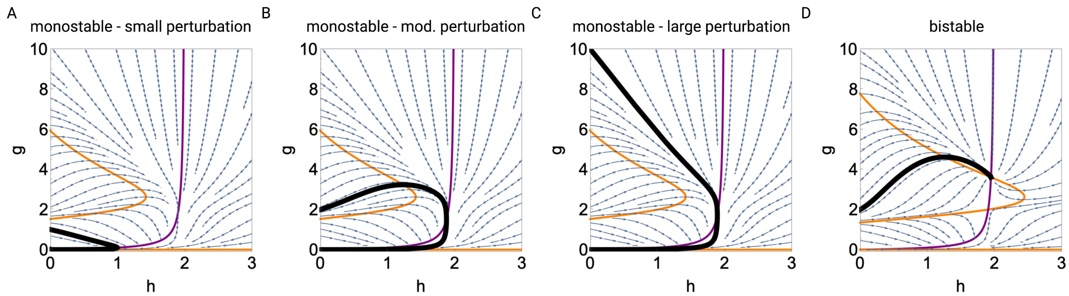

Figure S7: **Dynamical trajectories of the rate equations Eqs. (5,6) starting from various initial conditions. Related to Figure 2 and STAR Methods.** Initial condition were set with $h = 0$ and variable g in the monostable regime (panels A-C) and the bistable regime (panel D). Parameters are the same as that used in Figure S5 in the respective regimes with nullclines labelled using the same color scheme.

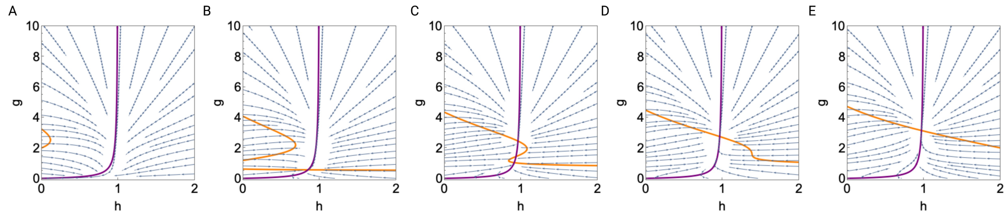

Figure S8: **Stream plot of Eqs. (5,6) and nullclines for $\dot{g} = 0$ (orange) and $\dot{h} = 0$ (purple) for nonzero production. Related to STAR Methods.** With setting (A) $I = 0$, (B) $I = 0.05$, (C) $I = 0.07$, (D) $I = 0.08$, and (E) $I = 0.1$. All other parameters are the same as Figure S5, with $V = 0.4$. Starting from the monostable regime (A), as I increases, the lower stable fixed point moves upwards and the upper orange nullcline is stretched towards the right (B). This may result in a saddle-node bifurcation (C), and in a bistable regime like the one described in previous figures, but with a non-zero lower stable fixed point. As d increases further, the lower stable / unstable fixed-point pair may coalesce and the system will undergo another saddle-node bifurcation, with only a stable silenced fixed-point (D,E).

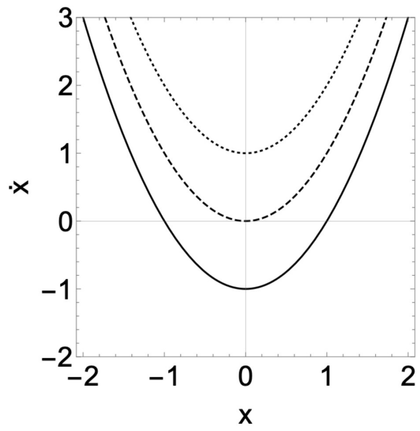

Figure S9: **Dynamical rates of the normal form $\dot{x} = x^2 - \mu$ for different values of μ. Related to STAR Methods.** Parameters were set as μ = 1 (solid), μ = 0 (dashed), and μ = −1 (dotted). When μ < 0 there are no finite fixed points and the dynamics converge to $x \to \infty$ (corresponding to the monostable regime of the TI model). When μ > 0 the system has a stable fixed point at $x = -\mu$ and an unstable fixed point at $x = \mu$ (corresponding to the bistable regime of the TI model). The case of μ = 0 corresponds to the critical regime in the TI model - as $\mu \to 0$ the dynamics of the system become delayed around x = 0.

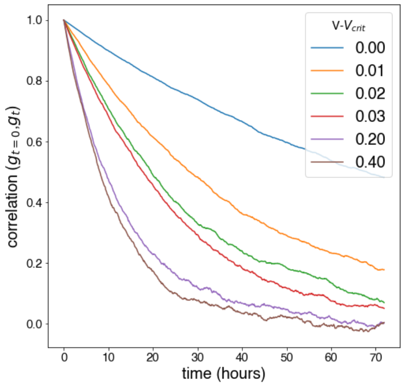

Figure S10: **Autocorrelation of individual sample paths. Related to STAR Methods.** Autocorrelation of sample paths near the critical point during a single generation, three generations after initiation of silencing, for different values of $V - V_{\mathrm{crit}}$, measured by the Pearson correlation between $g(t = 0)$ and $g(t = \mathrm{lag})$. To prevent trajectories from crossing the critical point, noise was reduced to $\sigma = 0.0001$. Other parameters and simulation details are identical to Figure S7, and the generation time τ is provided in Table 3.

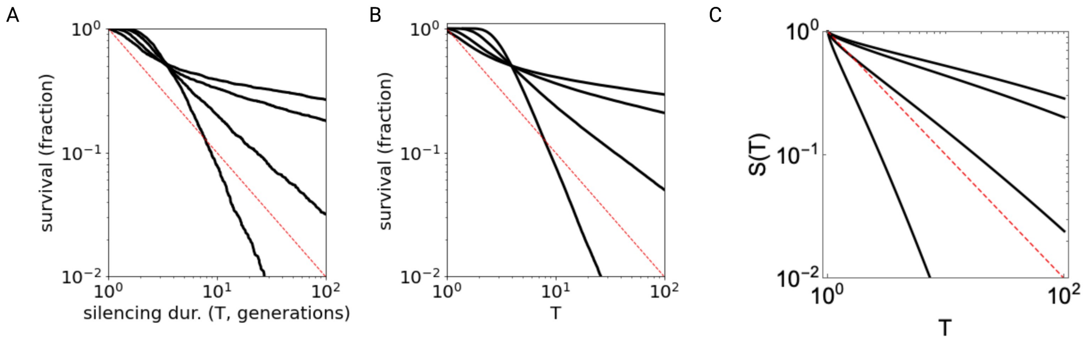

Figure S11: **Survival time distributions of gene silencing. Related to STAR Methods.** (A) Survival time distribution obtained from stochastic simulation of Eqs. (17,18), taking the parameters as in Figure S7, but setting $V \sim \text{Normal}(V_{\text{crit}}, b)$, with $b = 0.01, 0.02, 0.04, 0.06$ (lower to upper lines). Noise is set to $\sigma_1 = \sigma_2 = 0.03$. Trajectories start at $(g_0, h_0) = (3,0)$ and are considered silenced until the silencing metric $s(g,h)$ drops below the threshold s_m. This results in a distribution of survival times, which is depicted on a log-log plot. (B) The survival distribution of T calculated from Eq. (22), taking $\sigma = 2$ and drawing μ from a normal distribution $\mu \sim \text{Normal}(0,b)$ with $b = 1, 2, 4, 6$. (C) Predicted survival time distribution $S(T)$ as defined by Eqs. (28,29), setting $b = 0.01, 0.02, 0.04, 0.06$ (lower to upper lines). Note that this approximation, which captures the bistable portion of V, is appropriate only for the slope of tails of the survival distributions depicted in panel A. Here, we have set $A = 1$, $B = 482$. Adjusting A does not sensitively affect the outcome if $\ln T \gg \ln A$, while B is inferred from simulations of average silencing duration vs. $V - V_{\text{crit}}$. The dashed red line denotes t^{-1}. We note that, as the variation b increases, the tail of the survival distribution asymptotes towards a flat line parallel to the time axis.

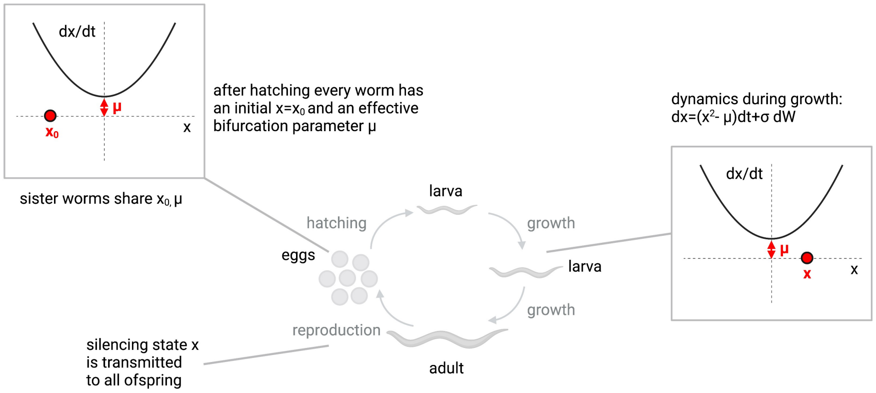

Figure S12: **Model overview. Related to STAR Methods.** Following hatching, each worm is characterized by a silencing coordinate x, as well as a bifurcation parameter μ. We assume that these parameters are largely shared between sister worms. The dynamics during growth from larva to adult are simulated according to Eq. (19) (adjusted in Eq. (30) to allow for de-silencing).

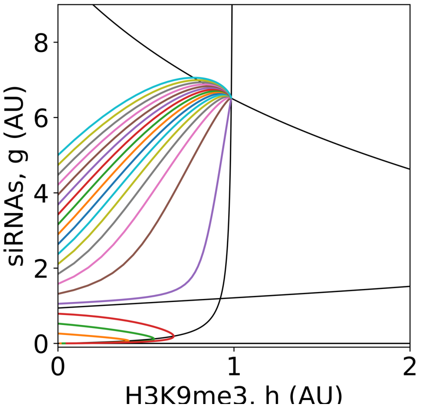

Figure S13: **TI Model simulations in the bistable regime, with different initial conditions. Related to STAR Methods.** One-generation simulations of the TI model in the bistable regime starting from $h = 0$ and a variable initial g, setting $V = 1$ (bistable regime), with all other parameters as in Table S1. Simulations are plotted as trajectories in phase-space.

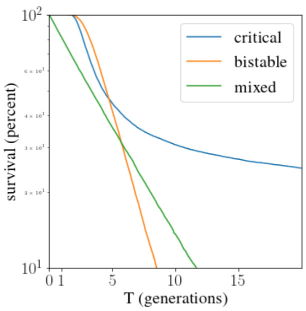

Figure S14: **Silencing duration survival distributions in different regimes (critical/bistable/monostable). Related to STAR Methods.** Predicted silencing duration survival distribution (plotted in log-linear scale) given variation in μ for the critical model ($\mu \sim N(-0.5, 1)$, $\sigma = 0.5$), bistable model ($\mu \sim N(10, 3)$, $\sigma = 8$), and the mixed model, with a switching probability γ (with $\gamma^{-1} \sim N(5, 1)$).

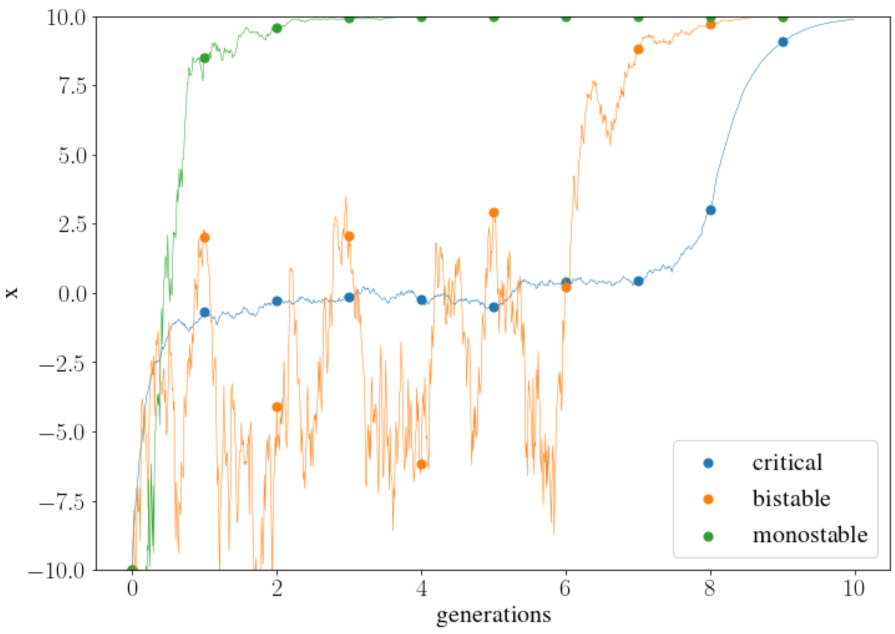

Figure S15: **Examples of dynamics of different regimes (critical/bistable/monostable). Related to STAR Methods.** Simulations of Eq. (30) for the critical regime ($\mu = -0.5$, $\sigma = 0.5$), bistable regime ($\mu = 10$, $\sigma = 8$), and monostable regime ($\mu = -100$, $\sigma = 10$). For all simulations we set $x_{\min} = 1$, $x_{\max} = 10$, $\gamma = 2$.

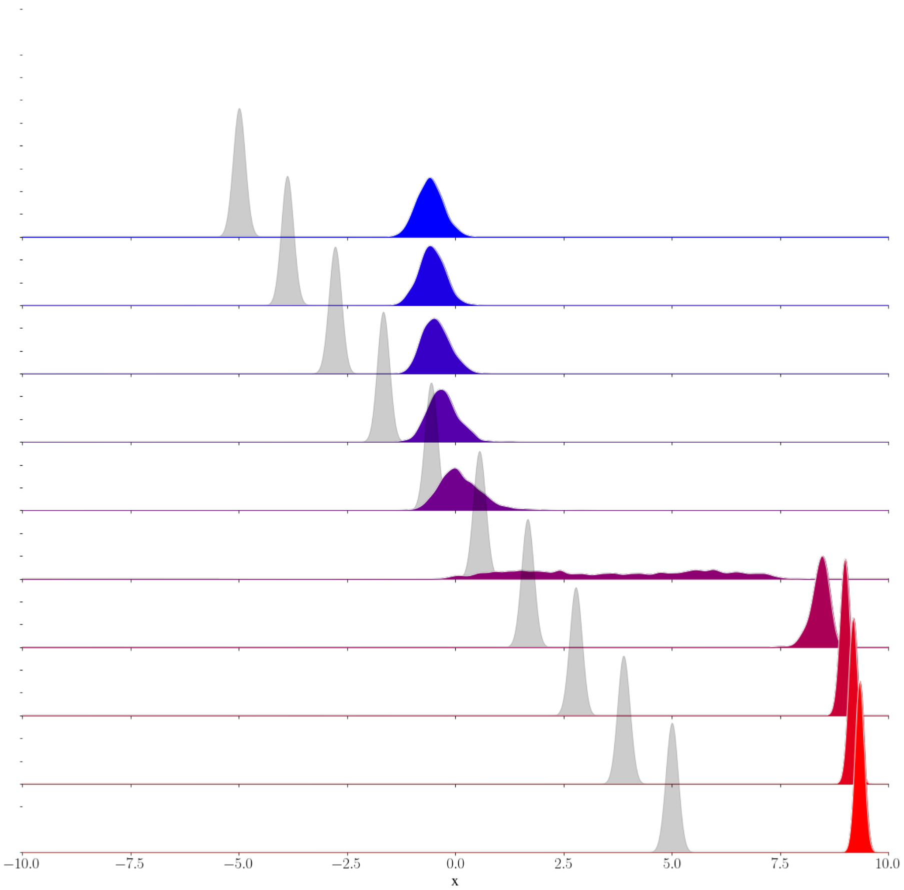

Figure S16: **Single generation dynamics, starting from different conditions, in the critical regime. Related to STAR Methods.** Single generation simulations of Eq. (30) for the critical regime ($\mu = 0$, $\sigma = 0.5$, other parameters set as in Figure S15). Each row depicts the distribution of x at the end of the generation (red-blue distributions) when the simulation is initialized with values sampled from the gray distribution, modelling the dynamics of sister worms that start from similar initial conditions.

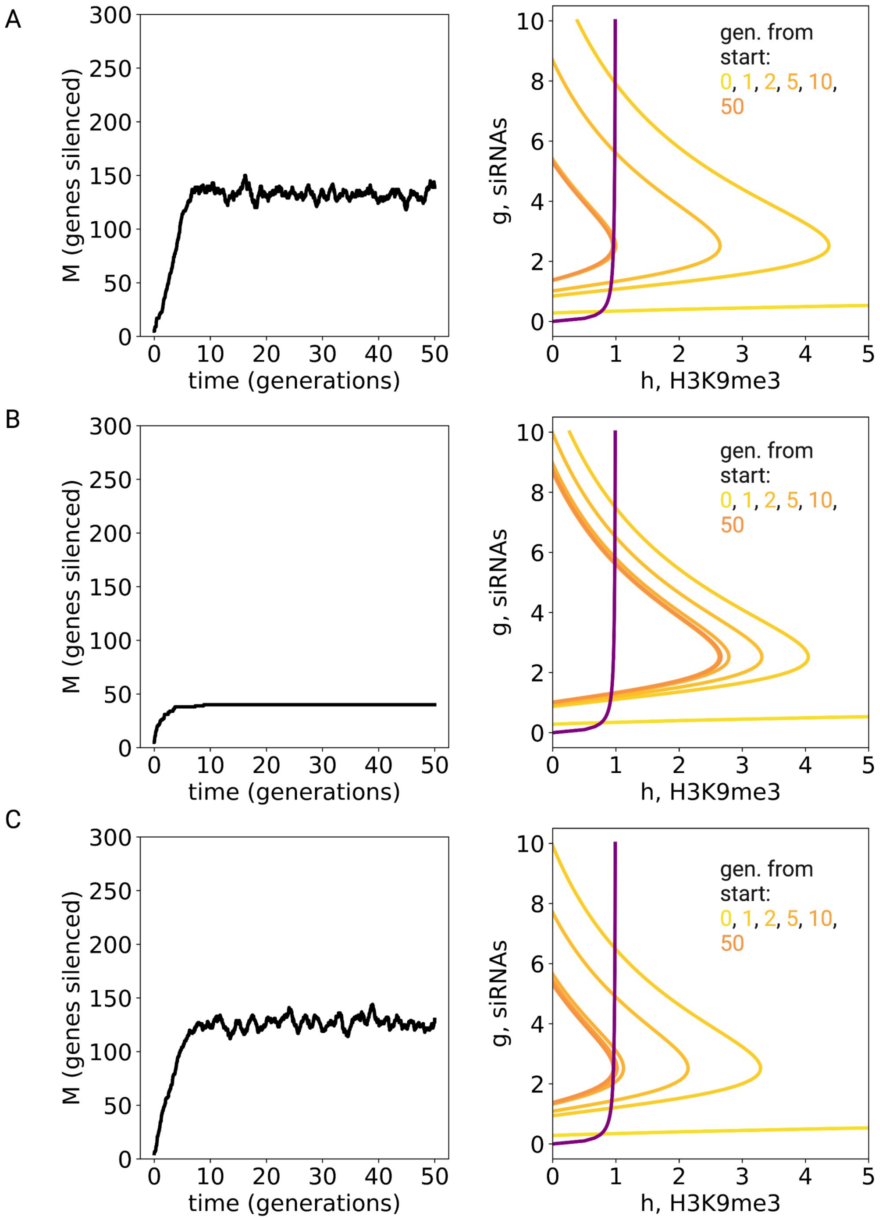

Figure S17: **TIC model simulations for finite pools of silenced genes. Related to Figure 4 and STAR Methods.** Stochastic simulations of the TIC model (as in Figure 4A,B), taking either (A) an infinite pool of genes that can be silenced, or (B) a finite pool of magnitude $N = 40$ or (C) $N = 400$. When all candidate genes become silenced (as in panel B), the system may stabilize in the bistable region.

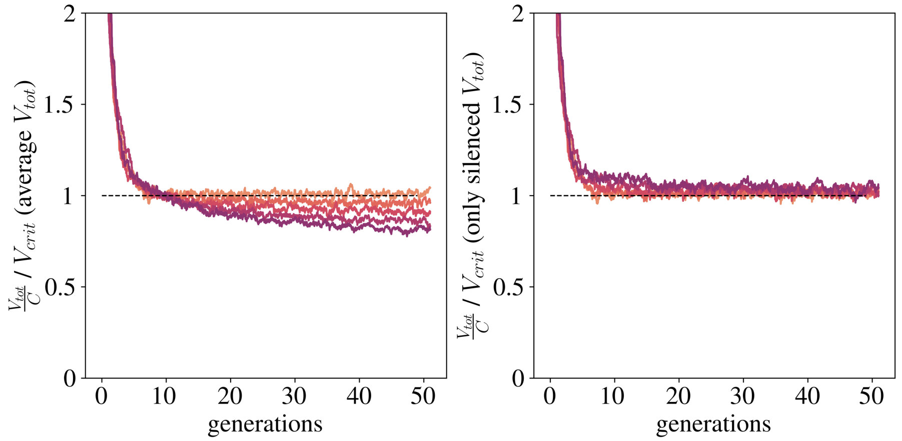

Figure S18: **TIC model with variation in V_{tot}. Related to Figure 4 and STAR Methods.** (A) Change in $\frac{V_{tot}}{C}$ as a function of generation number in the situation where memory population dynamics were simulated as in Figure 4 in the main text, but where V_{tot} for individual genes is drawn from a normally-distributed random variable with 0%, 5%, 10%, 15%, 20% standard deviation around the mean (blue, orange, green, red, and purple line respectively). (B) In all cases, the value of $\frac{V_{tot}}{C}$ of the silenced genes remains close to V_{crit}.

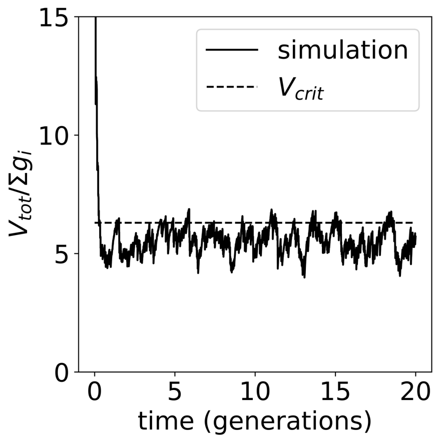

Figure S19: **Simulation of TIC model with pUGylation dynamics. Related to STAR Methods.** Simulation of Eqs. (42-45), taking all parameters as in Tables S1-S3, with the simulation starting with no silenced genes. The effective $\frac{V_{tot}}{\Sigma g_i}$ converges rapidly to the vicinity of the critical value.

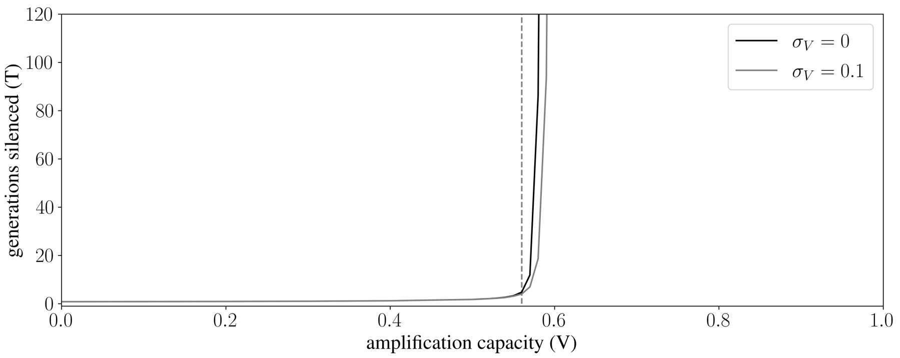

Figure S20: **Simulation of TI model with fluctuating V. Related to STAR Methods.** Simulation of Eqs. (17,18,49), taking all parameters as in Tables S1-S3, for variable V_0, setting $\gamma_V = 0.1$, and $\sigma_V = 0$ (dashed, no fluctuations) or $\sigma_V = 0.1$ (solid, fluctuations in V). The additional noise shifts the dependence of T on V_0 but the steep dependence around the critical point remains.

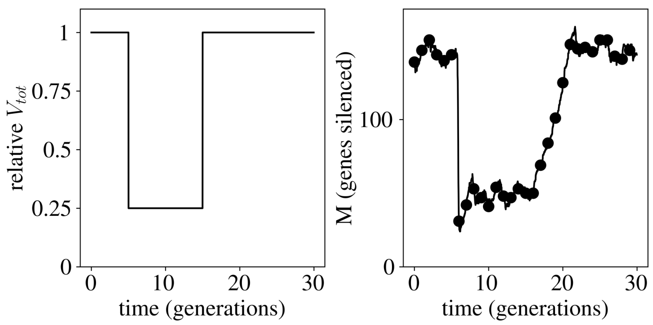

Figure S21: **Step changes in parameters result in asymmetric silencing dynamics in the TIC model. Related to STAR Methods.** A down-change in the silencing pool size M (such as due to a down-step in V_{tot}) is driven by a transition of the system to the monostable regime and thus has a fast timescale. An up-change in M, on the other hand, requires re-accumulation of silenced genes and thus has a longer timescale, on the order of T. This is evident by comparing the effect of down- and up-steps in V (left) on the silencing pool size M (right). Simulation parameters are provided in Tables S1-S3.

Parameter	Value	Units	Comment
$\boldsymbol{V}$	$0.45, 0.55, 0.8$	[AU] hr^{-1}	
$\boldsymbol{n}$	3	unitless	
$\boldsymbol{k_1}$	2	[AU]	
$\boldsymbol{k_2}$	2	[AU]	
$\boldsymbol{k_3}$	0.1	[AU]	
$\boldsymbol{\psi}$	0.1	[AU] hr^{-1}	
$\boldsymbol{\gamma_1}$	0.1	hr^{-1}	dilution timescale
$\boldsymbol{\gamma_2}$	0.1	hr^{-1}	dilution timescale
$\boldsymbol{\gamma_3}$	0.1	hr^{-1}	dilution timescale
$\boldsymbol{\gamma_4}$	0.1	hr^{-1}	dilution timescale
$\boldsymbol{I}$	0.3	[AU] hr^{-1}	dsRNA trigger
$\boldsymbol{I}$	0	[AU] hr^{-1}	no dsRNA trigger
$\boldsymbol{\sigma_1}$	0.02	$\mathrm{hr}^{-1/2}$	
$\boldsymbol{\sigma_2}$	0.02	$\mathrm{hr}^{-1/2}$	
$\boldsymbol{\sigma_3}$	0.02	$\mathrm{hr}^{-1/2}$	
$\boldsymbol{\sigma_4}$	0.02	$\mathrm{hr}^{-1/2}$	

Table S1: **TI model simulation parameters. Related to Figures 2-4.**

**Parameter**	**Value**	**Units**
$\boldsymbol{V_{tot}}$	200	[AU] hr^{-1}
$\boldsymbol{\lambda}$	20	events $\mathrm{generation}^{-1}$

Table S2: **Additional simulation parameters for the TIC model. Related to Figure 4.**

**Parameter**	**Value**	**Units**
$\boldsymbol{s}_{\mathrm{silenced}}$	0.5	[AU]
$\boldsymbol{s}_{\mathrm{select}}$	3	[AU]

Table S3: **Other simulation parameters. Related to Figures 3-4.**
